# Impact of Reduced Anthropogenic Emissions Associated With COVID‐19 Lockdown on PM_2.5_ Concentration and Canopy Urban Heat Island in Canada

**DOI:** 10.1029/2023GH000975

**Published:** 2025-02-01

**Authors:** Samaneh Ashraf, Francesco S. R. Pausata, Sylvie Leroyer, Robin Stevens, Rodrigo Munoz‐Alpizar

**Affiliations:** ^1^ Department of Chemistry, University of Montreal (UdeM) Montreal QC Canada; ^2^ Centre ESCER (Étude et la Simulation du Climat à l’Échelle Régionale) and GEOTOP (Research Centre in Earth System Dynamics), Department of Earth and Atmospheric Sciences, University of Quebec in Montreal (UQAM) Montreal QC Canada; ^3^ Meteorological Research Division Environment and Climate Change Canada Montreal QC Canada; ^4^ Climate Research Division Environment and Climate Change Canada Victoria BC Canada; ^5^ Meteorological Service of Canada, Environment and Climate Change Canada Montreal QC Canada

## Abstract

Extensive lockdowns during the COVID‐19 pandemic caused a remarkable decline in human activities that have influenced urban climate, especially air quality and urban heat islands. However, the impact of such changes on local climate based on long term ground‐level observations has hitherto not been investigated. Using air pollution measurements for the four major Canadian metropolitan areas (Toronto, Montreal, Vancouver, and Calgary), we find that PM_2.5_ markedly decreased during and after lockdowns with peak reduction ranging between 42% and 53% relative to the 2000–2019 reference period. Moreover, we show a substantial decline in canopy urban heat island intensity during lockdown and in the post lockdowns periods with peak reduction ranging between 0.7°C and 1.6°C in comparison with the 20‐year preceding period. The results of this study may provide insights for local policymakers to define the regulation strategies to facilitate air quality improvement in urban areas.

## Key Points

- The drastic reduction in human activity during COVID‐19 lockdown led to a significant decrease in both air pollution and the CUHI intensity
- The PM_2.5_ average was reduced up to almost 2 S.D. throughout the lockdown and after the lockdown period in comparison with the long‐term mean
- The CUHI intensity dropped up to almost 1.5 S.D. during both the lockdown and post‐lockdown periods in comparison with the historical mean

## 1 Introduction

Air pollution is considered the primary environmental cause of disease and premature death globally (Stanaway et al., 2018). The most prevalent contaminants found in the atmosphere, including particulate matter (PM) particularly PM_2.5_ with aerodynamic diameters ≤2.5 μm, ozone (O_3_), nitrogen oxides (NO and NO_2_), sulfur dioxide (SO_2_), and carbon monoxide (CO), negatively impact the respiratory and cardiovascular systems (Lelieveld et al., 2015; Rybarczyk & Zalakeviciute, 2021). Prolonged exposure to air pollution including PM_2.5_ and O_3_ is estimated to cause ∼8.8 million excess deaths annually (Burnett et al., 2018; Lelieveld et al., 2020), and approximately 4 million new cases of asthma in children are attributed to NO_2_ exposure (Achakulwisut et al., 2019).

The global outbreak and spread of COVID‐19 in early 2020 impacted urban environments significantly (Tian, An, et al., 2021; Tian, Cui, et al., 2021) and air quality as a result of extreme reductions in human activities (Berman & Ebisu, 2020). Understanding how air pollutant (e.g., NO_2_, O_3_, and PM_2.5_) levels have been changed during the COVID‐19 pandemic will give important insights into health effects and emission control (Berman & Ebisu, 2020; Gough & Anderson, 2022; Q. Liu et al., 2021; Mashayekhi et al., 2021). The variation of fine particulate matter (PM_2.5_), which is the main environmental risk factor for mortality worldwide, is of particular interest (Hammer et al., 2021). Numerous studies around the world have utilized satellite data and/or model simulations to evaluate the variations in main air pollutants levels after the outbreak of COVID‐19 pandemic and during periods of city lockdowns (Bauwens et al., 2020; Ding et al., 2020; El Kenawy et al., 2021; C. Fan et al., 2020; Goldberg et al., 2020; Huang et al., 2021; Le et al., 2020; Li et al., 2020; Muhammad et al., 2020; Venter et al., 2020; Wang et al., 2020). Despite the usefulness of remote sensing in representing a broad overview of air pollution levels, it is imperative to supplement this data with in‐situ measurements, as the use of ground‐level observation holds a fundamental significance in confirming and consolidating the pollution trends (Bechle et al., 2013). While satellites offer global coverage, they have certain drawbacks, such as limited historical data and inaccuracies in reflecting ground‐level pollutant levels (Venter et al., 2020). Discrepancies in pollutant trends between ground and satellite measurements, caused by factors like dilution and meteorological effects, further support the preference for ground‐based data. Ultimately, ground‐based measurements are considered more relevant to public health, emphasizing their importance in quantifying air pollution impacts accurately (Venter et al., 2020). Ground‐level observations provide valuable standards for pollutant concentrations and serve as a means for regulatory compliance. These observations can be used to assess changes in air pollution, particularly in areas where extensive monitoring systems are established (Berman & Ebisu, 2020). While there are several studies to evaluate variation of air pollutants due to COVID‐19 lockdowns by using ground‐based observational data (Adams, 2020; Berman & Ebisu, 2020; Fan et al., 2021; Liu et al., 2021; Rybarczyk & Zalakeviciute, 2021), such studies adopted a very short reference period ranging from only several months prior to the COVID‐19 quarantine to a maximum of 5 years. Such short reference periods hamper a proper quantification of how much of those observed changes in pollution during 2020 were associated with COVID‐19 and how much were due to interannual fluctuations. Therefore, there is a lack of comprehensive analysis from prolonged ground‐based observations at city scale that may be used by local policymakers.

Another impact of COVID‐19 lockdowns on the environment is its potential effect on the urban heat island (UHI) (Jallu et al., 2022; Parida et al., 2021). The UHI phenomenon refers to the tendency of temperatures to be higher in urban areas compared to surrounding rural areas (Liu et al., 2022; Zhou et al., 2018). The UHI is a significant human‐induced change to the environment and is primarily resulting from changes in the form, structure, and characteristics of the surface when natural landscapes are converted into urban areas (Cao et al., 2016; Lazzarini et al., 2015; Logan et al., 2020; Zhou et al., 2018). In particular, low‐reflectivity materials such as concrete used in cities trap radiation, which is then released at night as sensible heat (Biggart et al., 2020; Estoque et al., 2017). Urban geometry accentuates this energy trapping due to the multi‐reflection of solar radiation (Oke, 1988; Ryu & Baik, 2012). Impermeable surfaces used in cities alter the water budget by reducing infiltration and evapotranspiration and increasing surface runoff (Strohbach et al., 2019). Notably the heat produced by human activities also affects the UHI (Stewart & Oke, 2012). Urban areas are typically more densely populated and produce higher levels of anthropogenic heat release compared to surrounding rural areas, mainly as a result of road transportation, commercial and industrial activities, domestic heating and cooling, and human metabolism (Allen et al., 2011; Z. Liu et al., 2022). The UHI phenomenon represents one of the most significant hazards that directly impact health and quality of life and will have far‐reaching effects on society and the infrastructure that supports civilization (Shahmohamadi et al., 2011). There are two primary approaches to measuring and quantifying UHI: (a) Canopy‐layer UHI (CUHI), which is based on temperature at the canopy layer, and (b) Surface UHI (SUHI), which is based on surface temperatures (Pena Acosta et al., 2023).

Urban pollution and the UHI are both symptoms of the intense changes due to human settlement, energy consumption, transportation, industry, and the strain on natural resources resulting from rapid urbanization and population growth (Ulpiani, 2021). The COVID‐19 virus, which resulted in one of the most severe slowdowns in human activities in recent decades, was first reported in Canada in January 2020. By July 2022, the outbreak of this disease had infected ∼4 million people and caused ∼41,000 deaths across the country (Figures S1 and S2 in Supporting Information S1). Here we use ground‐level measurements from air quality stations of the four major Canadian metropolitan areas (located in the provinces with the most confirmed COVID‐19 cases) to quantify the effect of lockdown as a result of the COVID‐19 pandemic on the pollution level of the local urban environment. Moreover, using the in‐situ surface air temperature (SAT) observations within and outside the urban areas, this study attempts to investigate the variation of canopy UHI intensity associated with the reduced human activity during COVID‐19 lockdowns. When evaluating the impact of the COVID‐19 pandemic on urban environments, it is crucial to consider both anthropogenic and natural atmospheric processes influencing air quality. However, previous studies often neglected the impact of meteorology on air pollution dynamics. In our study, we have addressed this aspect by integrating meteorological parameters into our analysis and explicitly acknowledging their potential influence when examining the observed changes in air pollution and UHI intensity during the COVID‐19 pandemic. By considering the interplay between human activities and natural atmospheric phenomena, we can assess the physical contributors to changes in air quality and draw more accurate conclusions about their effects on urban environments.

## 2 Materials and Methods

### 2.1 Study Area

**Figure 1:**
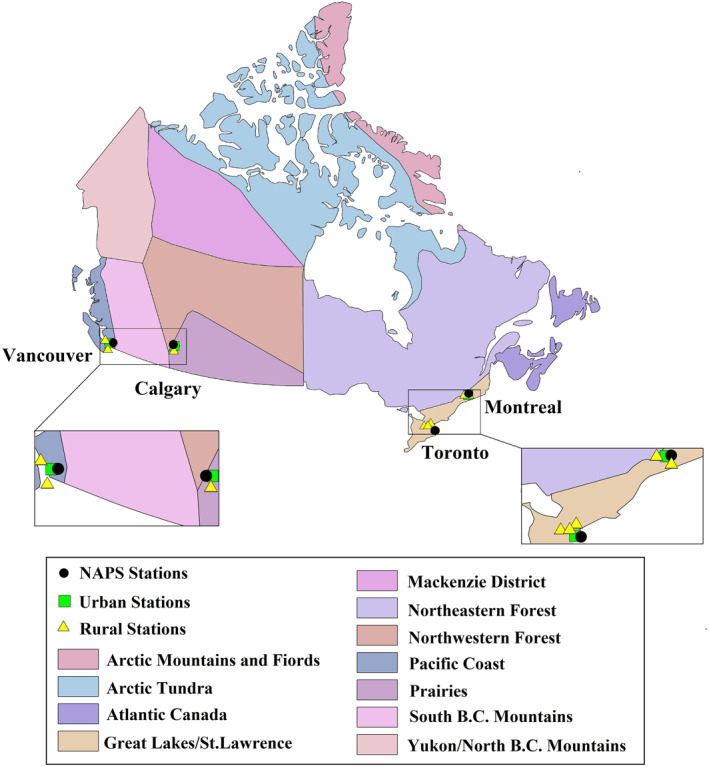
Four largest Canadian metropolitan census areas, along with the physiographic regions.

The four largest metropolitan areas in Canada, Toronto in Ontario, Montreal in Quebec, Vancouver in British Columbia and Calgary in Alberta, were considered in this study (Figure 1 and Table S1 in Supporting Information S1). The population of the Greater areas of these cities (on 9 February 2022) ranged between 1,481,806 inhabitants in Calgary to 6,202,225 in Toronto (Table S1 in Supporting Information S1) (https://www12.statcan.gc.ca/census‐recensement/2021/rt‐td/population‐eng.cfm).

### 2.2 Air Pollution Data and Statistical Analysis

Air pollution data (PM_2.5_, O_3_, NO_2_, SO_2_) from urban stations of each of the four cities (Figure 1 and Table S2 in Supporting Information S1) from 2000 to 2021 were obtained from the National Air Pollution Surveillance (NAPS) network that is the main source of ambient air quality data in Canada (https://data‐donnees.az.ec.gc.ca/data/air/monitor/national‐air‐pollution‐surveillance‐naps‐program/Data‐Donnees/?lang=en). The objective of the NAPS program is to provide precise, long‐term air quality data of a uniform standard throughout Canada, which is managed jointly by Environment and Climate Change Canada (ECCC) and networks of provincial, territorial, and regional governments (NAPS, 2021). In this research, we focus exclusively on variation of PM_2.5_ concentrations as other pollutants underwent long‐term trends and the COVID‐19 effect is consequently more difficult to detect (Figures S3–S6 vs. Figure S7 and Tables S3–S6 in Supporting Information S1). The relative change (percentage change) and the absolute change in the mean concentration (μg/m3) of PM_2.5_ were compared between the pre‐lockdown period (January to mid‐March), lockdown period (mid‐March to early June), and post‐lockdown period (late‐June to December) of 2020 and the preceding 20‐year period (2000–2019) for the four largest Canadian census metropolitan areas.

### 2.3 Ground‐Based Meteorological Data

We used daily measurements of maximum and minimum SAT, precipitation and wind speed, from ground‐based automatic stations distributed across each city (Figure 1 and Table S7 in Supporting Information S1) provided by the ECCC (https://climate.weather.gc.ca/index_e.html). The urban stations selected for this study are mostly situated in areas with a high percentage of impervious surfaces, whereas rural stations are generally located in grassland and cropland areas (Figure S8 in Supporting Information S1).

### 2.4 Quantifying the Canopy Urban Heat Island (CUHI) Intensity

In our study, we focus on the canopy layer UHI, which is more relevant to public health (Oke et al., 2017). We determine the variation of the canopy UHI as the temperature difference between urban and rural areas using the simplified urban‐extent algorithm (Chakraborty & Lee, 2019). Urban areas are typically characterized by higher temperature compared to the surrounding rural regions and the warmest part is usually located in the city center. It is ideal for the non‐urban reference point to be in a rural area with natural vegetation cover far enough from the city to avoid its influence, but close enough to have a similar microclimate (Martin‐Vide et al., 2015). In this study, the urban stations are situated in the downtown areas of each city, while the rural stations are selected in grassland regions approximately 30–50 km away from the city center. The intensities of canopy UHI (CUHI) were calculated based on observed monthly mean temperature with the following equation (Liu et al., 2022):

(1) $$\text{CUHI}\left(t\right)=\bar{{\text{SAT}}_{\text{urban}}\left(t\right)}-\bar{{\text{SAT}}_{\text{rural}}\left(t\right)}$$

The variations in CUHI during lockdown periods were evaluated by comparing the mean CUHI in the COVID‐19 outbreak year (2020) with the mean CUHI in the reference period. Here, we selected a 20‐year reference period (2000–2019):

(2) ∆CUHI(t)=CUHI(2020)−CUHI(referenceperiod)

Where ${∆I}_{c}\left(t\right)$ is the monthly anomaly in monthly CUHI between the lockdown year (2020) and the reference period (2000–2019).

### 2.5 Assessment of Changes in Meteorology

To assess the potential impact of weather anomalies on PM_2.5_ and CUHI, we quantify the number of rainy days (precipitation >1 mm/day) and number of windy days (maximum wind speed ≥50 km/hr) in 2020 in comparison with the preceding reference period. The reference periods for each city vary depending on data availability. For precipitation data, Toronto's reference period is from 2003 to 2020, while Montreal, Vancouver, and Calgary's reference periods are from 2000 to 2020. However, based on wind data, Montreal's reference period is from 2000 to 2020, for Toronto and Vancouver is from 2007 to 2020, and Calgary's reference period is 2008–2020. We focus on anomalies in rain and wind as they are associated with wet and dry deposition, respectively, and are also a good indicator for stronger or weaker UHI.

### 2.6 Quantifying Meteorology Effect Using GEOS‐Chem

To validate the observational analysis in the previous section, we conducted a set of sensitivity simulations using the GEOS‐Chem chemical transport model version 14.1.1 (https://doi.org/10.5281/zenodo.7696632, Bey et al., 2001; Park et al., 2004). GEOS‐Chem has previously been shown to resolve well the variability in PM_2.5_ concentrations across North America and for Quebec specifically (Kim et al., 2015; Stevens et al., 2024). GEOS‐Chem is driven by assimilated meteorology from the Modern‐Era Retrospective analysis for Research and Applications, Version 2 (MERRA‐2) data set produced by the NASA Global Modeling and Assimilation Office (https://gmao.gsfc.nasa.gov/reanalysis/MERRA‐2/citing_MERRA‐2/). We generate boundary conditions using a global simulation at a resolution of 2° latitude x 2.5° longitude. Subsequently, we employ a nested grid at a higher resolution of 0.5° latitude × 0.625° longitude, covering the geographical extent from 35° N to 70° N and 142° W to 52° W. This grid encompasses the entirety of Canada and significant portions of the United States known to be sources of pollution affecting Canada. Biomass‐burning emissions are simulated from the Global Fire Emissions Database version 4 (GFED4) (Giglio et al., 2013). Anthropogenic emissions from Canada are provided by the Air Pollutant Emissions Inventory (APEI), recently processed for use in chemical transport models. Anthropogenic emissions from the US are provided by the National Emissions Inventory for 2016 with annual scaling factors to account for changes in emissions relative to 2016 (Stevens et al., 2024). Shipping emissions are provided by the Community Emissions Data System version 2 (Hoesly et al., 2018) and aircraft emissions are provided by the Aviation Emissions Inventory Code (Simone et al., 2013; Stettler et al., 2011). Further details on the model configuration are provided in Text S1 in Supporting Information S1. Additionally, the comparison of the GEOS‐Chem PM_2.5_ simulations with observations is available in Table S8 in Supporting Information S1.

To examine the impact of meteorological conditions on changes in PM_2.5_ during the COVID‐19 period in 2020, we conducted ten base‐case scenario simulations (set 1) for all Mays between 2010 and 2019 (as May 2020 showed the most significant decrease in PM_2.5_). We then carried out two additional sets of 10 simulations for May 2020: one using anthropogenic emissions (set 2) and another using both anthropogenic and natural emissions (set 3) from 2010 to 2019. This approach allowed us to determine whether unusual meteorological conditions in May 2020 contributed to the observed reduction in PM_2.5_ levels during that month. The significance of the difference between the 2020 simulations (set 3) and the baseline simulations (set 1) was evaluated using the Student's *t*‐test.

## 3 Results

### 3.1 Change in Urban Air Pollution

**Figure 2:**
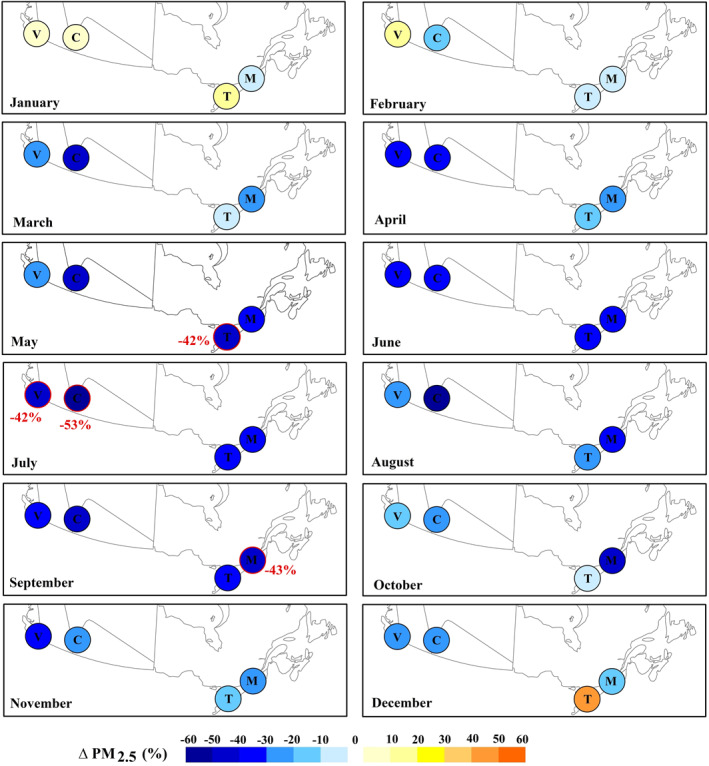
Percent change of monthly average of PM_2.5_ in 2020 relative to the 20‐year‐period (2000–2019) for Toronto (T), Montreal (M), Vancouver (V) and Calgary (C). The red values in the map represents the most significant reduction of PM_2.5_ relative to the reference period.

A decrease in PM_2.5_ from March to November 2020 can be seen for all four cities relative to the reference period (Figures 2 and S9 in Supporting Information S1). In particular, the most significant reduction of PM_2.5_ relative to the preceding 20‐year period (2000–2019) was observed in May in Toronto (∼−42%), in September in Montreal (∼−43%) and in July in Vancouver (∼−42%) and Calgary (∼−53%).

**Figure 3:**
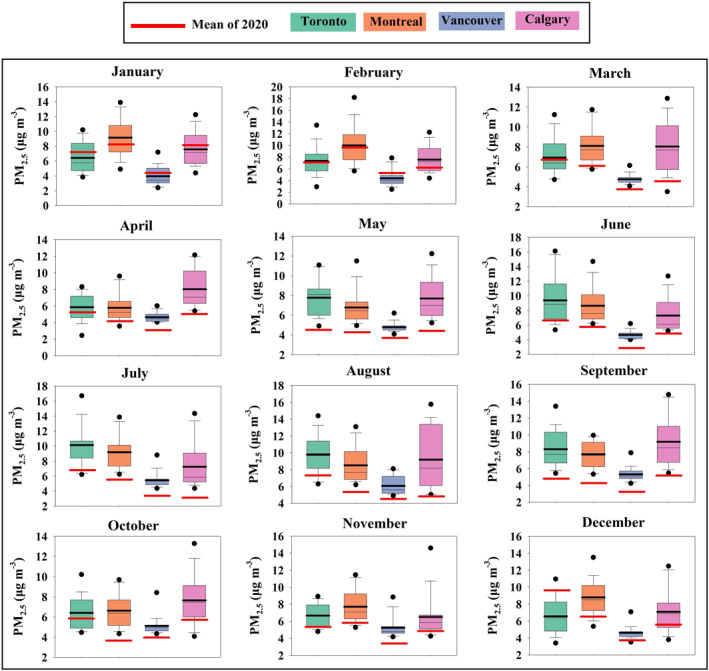
Comparison of monthly level of PM_2.5_ (μg/m^−3^) for Toronto, Montreal, Vancouver and Calgary in 2020 with the preceding 20‐year‐period (2000–2019). The lowest and highest points (the boundary of the lower and upper whisker) are the minimum and the maximum value of the data set. The box is drawn from first quartile to third quartile with a horizontal line drawn in the middle to denote the median. Black bold line in each box is the mean of the data set. Black dots are outliers. Red line in each box is the mean of 2020.

PM_2.5_ averaged concentrations in 2020 for Toronto, the most populated city in Canada, started decreasing in March, the first month of lockdown, with a slight—not significant—change of about −0.2 μg/m^3^ relative to the average of the reference period (2000–2019) of ∼6.9 μg/m^3^. The anomaly kept increasing and peaked in May with a significant reduction of 3.3 μg/m^3^, almost two standard deviations (S.D.) away from the mean, corresponding to a 42% reduction in the PM_2.5_ concentration compared to the climatological monthly mean of 7.8 μg/m^3^ (Figure 3‐Green boxes). The average PM_2.5_ concentration in 2020 for Montreal, the second largest city in Canada, began to significantly decrease in April with a change of about −1.7 μg/m^3^ (∼1 S.D.) relative to the average of the reference period (∼5.8 μg/m^3^). The decline of PM_2.5_ concentrations continued and reached its peak reduction of about 3.3 μg/m^3^ in September 2020, corresponding to more than twice the S.D. and about 43% decrease relative to the reference period (Figure 3‐Orange boxes). For Vancouver, the westernmost Canadian city, the PM_2.5_ averaged concentrations in 2020 also started to decrease in March, the first month of lockdown, with a significant variation of around −1 μg/m^3^ (∼2 S.D.) relative to the reference mean of about 4.8 μg/m^3^. Then in July this city experienced the largest significant reduction of 2.3 μg/m^3^, more than 2 S.D. away from the mean, corresponding to a value about 42% lower than the climatological monthly mean of about 5.4 μg/m^3^ (Figure 3c‐Blue boxes). The decrease of PM_2.5_ in Calgary, the smallest—albeit most polluted—metropolitan area in this study, began in March with a remarkable change of about −3.5 μg/m^3^ (S.D. = 2.6 μg/m^3^) relative to the long‐term mean (2000–2019) of ∼8 μg/m^3^ in 2000–2019. The largest amount of decrease for this city was in July 2020 with a reduction of around 4.9 μg/m^3^ (S.D. = 3.3 μg/m^3^) or almost a 53% decrease compared to the reference period of about 9.2 μg/m^3^ (Figure 3‐Pink boxes).

### 3.2 Change in Canopy Urban Heat Island (CUHI)

Urban heat island is most noticeable during winter and summer (Table S9 in Supporting Information S1) across Canada. More specifically, the largest difference in historical monthly mean temperature between the urban and rural stations is 3.6°C, 1.8°C, and 1.6°C in winter for Toronto, Montreal, and Calgary, respectively. For Vancouver, the available rural stations with long‐term data availability are located on the islands west of Vancouver, that is, under a significantly different microclimate with cooler summers and warmer winters. Therefore, the estimation of the absolute CUHI for Vancouver using those rural stations could be misleading. However, in this study we are looking at relative anomalies and therefore we can still gain insights on possible relative changes in CUHI in Vancouver due to the pandemic.

**Figure 4:**
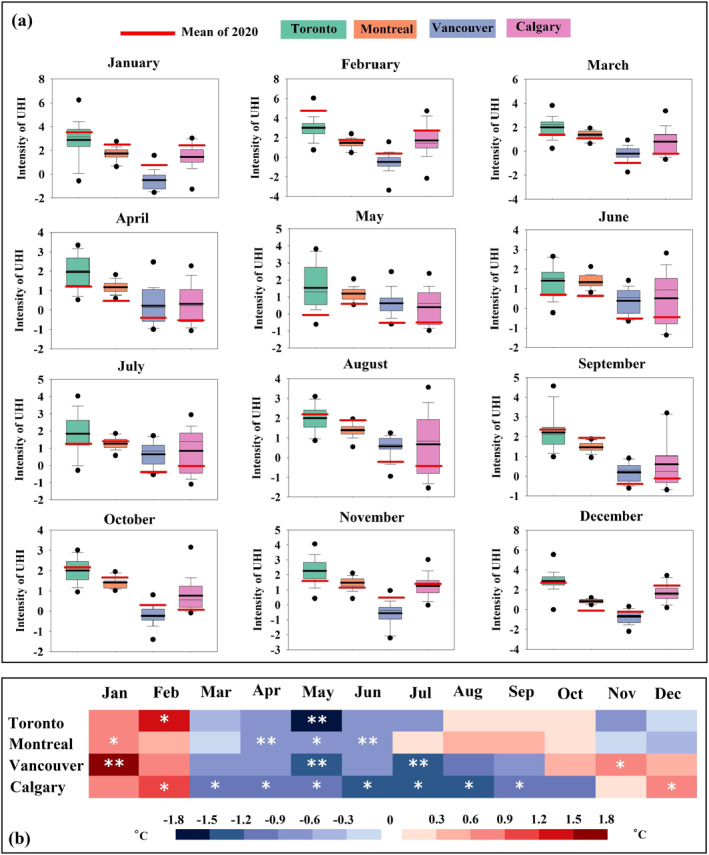
(a) Differences of mean temperature (intensity of urban heat island (UHI)) in urban and average of rural stations of each city and (b) variations in UHI intensity in 2020 relative to preceding 20‐year‐period (2000–2019) based on difference of mean temperature in urban and average of rural stations. The asterisks * and ** present observed change is greater than one and two standard deviations of the mean respectively.

During lockdown and in the post lockdown periods of 2020, negative CUHI anomalies (ΔI_c_) in comparison with pre‐lockdown periods appear in all cities based on mean, minimum and maximum temperatures (Figure 4 and Figures S10–S18 in Supporting Information S1).

For Toronto, the largest Canadian city, the most remarkable reduction of CUHI intensity derived from mean temperature (by considering the average of all rural stations) in 2020 relative to the reference period (2000–2019) was observed in May, around 1.6°C (one S.D. = 1.3°C) which is about 107% less than the climatological monthly mean of about 1.5°C (Figure 4a‐Green boxes and Figure 4b). For Montreal, the second populated city in Canada, the most negative monthly anomaly (ΔIc) registered (based on mean temperature) was of about 0.7°C (one S.D. = 0.3°C) in June 2020. That is ∼54% lower than the average of 2000–2019 of about 1.3°C (Figure 4a‐Orange boxes and Figure 4b). For Vancouver, the most western Canadian city, the largest amount of reduction in the CUHI intensity (based on mean temperature) relative to the average of the last two decades (2000–2019) was observed in May 2020. This change is about 1.2°C (one S.D. = 0.7°C), almost 200% lower than the mean of the reference period (0.6°C) (Figure 4a‐blue boxes and Figure 4b). In Calgary, the smallest metropolitan areas, the reduction in ΔIc (based on mean temperature) reached its peak in August 2020: about 1.4°C (one S.D. = 1.3°C). That is 200% less than the mean of 2000–2019 (0.7°C) (Figure 4a‐blue boxes and Figure 4b).

The most remarkable reduction of CUHI intensity based on maximum temperature (by considering all rural stations) in 2020 relative to the preceding 20‐year‐period (2000–2019) was observed in May for Toronto at around 2°C (one S.D. = 1.4°C) (Figures S12 and S18 in Supporting Information S1), in April for Montreal at around 0.7°C (one S.D. = 0.3°C) (Figures S13 and S18 in Supporting Information S1), in April for Vancouver at ∼1.7°C (one S.D. = 1.3°C) (Figures S16 and S18 in Supporting Information S1) and in July for Calgary at around 1.8°C (one S.D. = 1.3°C) (Figures S17 and S18 in Supporting Information S1). The most notable reduction of ΔI_c_ based on minimum temperature (among all rural stations) in 2020 in comparison with the mean long term climatological period (2000–2019) was recorded in March at around 1.7°C (one S.D. = 1.2°C) for Toronto (Figures S10 and S18 in Supporting Information S1), in June at around 1.7°C (one S.D. = 1.3°C; Figures S14 and S18 in Supporting Information S1) for Montreal, in July at around 1.8 (one S.D. = 1.2°C; Figures S16 and S18 in Supporting Information S1) for Vancouver and in October at around 2°C (one S.D. = 1.3°C) for Calgary (Figures S17 and S18 in Supporting Information S1).

### 3.3 Potential Impact of Weather Anomalies on Air Pollution and CUHI

**Figure 5:**
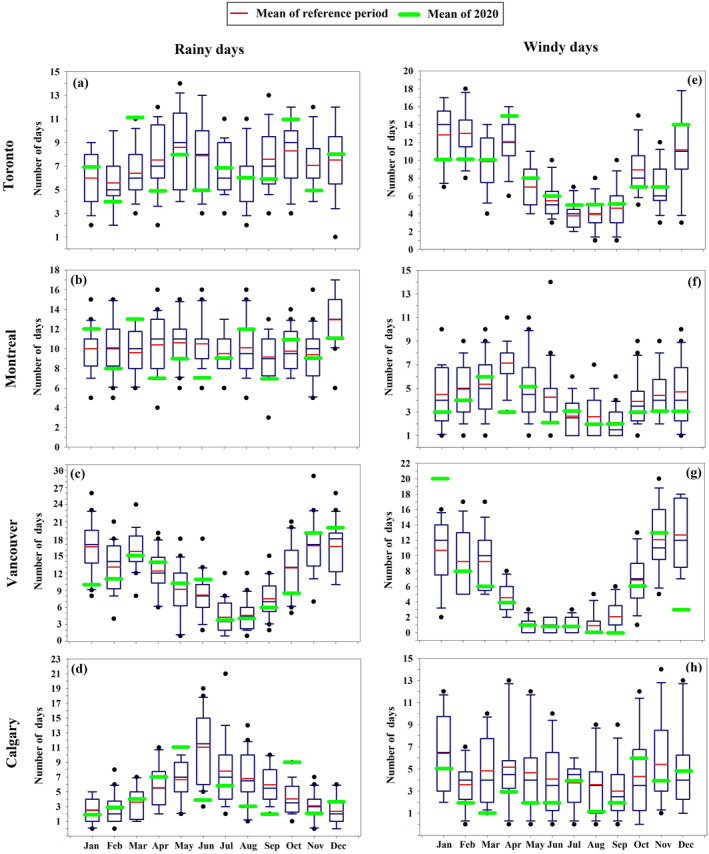
Box plot of (a–d) number of days with precipitation >1 mm and (e–h) number of days with speed of max Gust ≥50 km/hr in 2020 in comparison with the reference period, for urban stations within each city. The lowest and highest points (the boundary of the lower and upper whisker) are the minimum and the maximum value of the data set. The box is drawn from first quartile to third quartile with a horizontal line drawn in the middle to denote the median. Black dots are outliers.

Weather conditions largely shape pollutant concentrations at the surface (Shi et al., 2021) as they control atmospheric PM deposition as well as the intensity of the CUHI. Rainfall, snowfall, fog, etc., effectively remove PM from the atmosphere through the so‐called wet deposition, improving air quality (Tian, An, et al., 2021; Tian, Cui, et al., 2021). High wind speed facilitates the deposition of pollutants by increasing the probability of particles to impact surfaces, such as the ground, plants, buildings and therefore removing them from the atmosphere (dry deposition) (Briz‐Redón et al., 2021). Precipitation and high wind speed also reduced the intensity of the CUHI by increasing the vertical and horizontal mixing in the atmospheric boundary layer. On the other hand, stable conditions and low wind speed are in general associated with pollutant accumulation and increased CUHI. Here, we investigate whether the observed changes in PM_2.5_ concentrations and CUHI observed in 2020 can be attributed to anomalous rainfall and wind speed conditions (Figure 5).

Our analysis shows that in general the number of wet days in 2020 were below or around the average of their historical monthly mean for all metropolitan areas (Figures 5a–5d), which cannot justify the observed reduction in PM concentrations and in intensity of the CUHI. However, an unusually high number of wet days was observed in Toronto and Montreal in March of 2020 (∼72% and 51% more than the climatological mean respectively, Figures 5a and 5b and Table S10 in Supporting Information S1) and for Calgary in May and October of 2020 (∼65% and 122% more than the mean of the reference period respectively; Figure 5d and Table S10 in Supporting Information S1). Therefore, the slight and non‐significant reduction of PM_2.5_ concentration level (Figures 2 and 3) and CUHI intensity (Figure 4) in March for Montreal and Toronto and in October for Calgary may also be related to the higher than usual number of rainy days (Figures 5a and 5d) rather than in reduced human activities due to lockdown. The significant reduction of PM_2.5_ concentrations (Figure 3) and CUHI (Figure 4) intensity recorded in May for Calgary were likely a combination of the extremely high number of rainy days and the lockdown effect.

Comparison between the number of days with a max wind gust ≥50 km/hr in 2020 and those in the reference period (Figures 5e–5h and Table S10 in Supporting Information S1), indicate that all metropolitan areas of this study did not experience particularly more extreme wind events than usual during lockdown and post lockdown periods of 2020.

**Figure 6:**
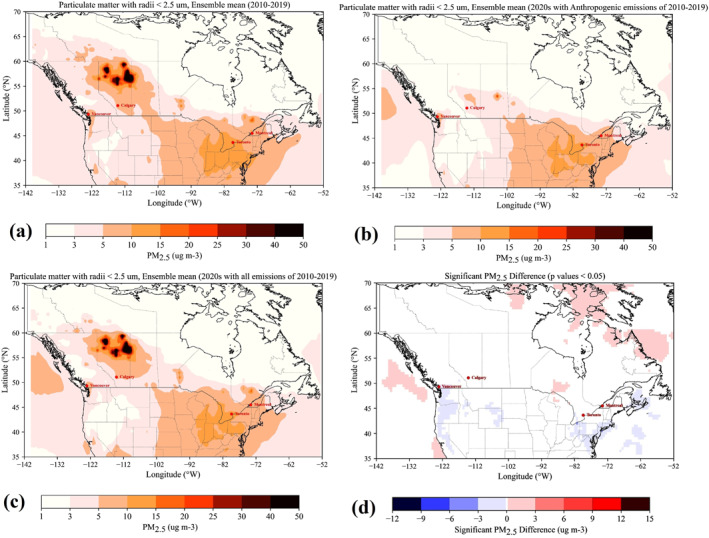
(a) Ensemble mean of GEOS‐Chem simulations of PM_2.5_ from 2010 to 2019. (b) Ensemble mean of GEOS‐Chem simulations for 2020 using anthropogenic emissions from the previous 10 years (2010–2019). (c) Ensemble mean of GEOS‐Chem simulations for 2020 using all emissions (anthropogenic and natural) from the previous 10 years (2010–2019). (d) Significant differences between 2020 simulations (using all emissions) and the 2010–2019 period (panel c minus panel a). Only changes significant at the 95% confidence level are shown.

To unequivocally assess whether the reduction in PM_2.5_ concentrations observed in May 2020 was due to reduced human activities or simply a result of changes in weather conditions, we conducted three sets of simulations using GEOS‐Chem. In the first set, we conducted baseline simulations using both natural and anthropogenic emissions, along with weather conditions, from each May between 2010 and 2019 (Figure S19_left panel in Supporting Information S1). In the second set, we performed 10 experiments using meteorological conditions and natural emissions from May 2020, combined with anthropogenic emissions from each of the preceding 10 years (2010–2019) (Figure S19_middle panel in Supporting Information S1). In the third set, we conducted 10 experiments using meteorological conditions from May 2020, combined with all emissions (anthropogenic and natural) from each of the preceding 10 years (2010–2019) (Figure S19_right panel in Supporting Information S1). By comparing the first and second sets of ensemble simulations (Figures 6a and 6b), we can identify variations in PM_2.5_ concentrations that were due to changes in both natural emissions and weather conditions in May 2020. Furthermore, by comparing the first and third sets of ensemble simulations (Figures 6a and 6c), we can isolate variations in PM_2.5_ concentrations that were solely due to changes in meteorological conditions in May 2020.

Our results indicate that the large observed anomalies in baseline period, particularly in northern Alberta, were due to wildfire emissions (natural emissions) that were not present in 2020 (compare Figures 6a and 6c, and Figure S19 in Supporting Information S1). Additionally, there is no significant change in PM_2.5_ concentrations (Figure 6d), supporting the conclusion that the changes observed in PM_2.5_ concentrations in major Canadian cities during the pandemic were indeed caused by emission reductions. Meteorological conditions, therefore, were not particularly anomalous enough to justify the large observed decrease in both PM_2.5_ concentration levels and CUHI intensity across the four cities analyzed in this study. Hence, the reductions in PM_2.5_ and CUHI presented in this study are largely attributable to the COVID‐19 lockdowns and the consequent decrease in human activities.

## 4 Discussion

In this study we investigate the variation of urban pollution and UHI in four largest metropolitan areas across Canada associated with the COVID‐19 lockdowns that led to the most intense slowdown in human activities in recent decades. Our finding shows that levels of PM_2.5_ concentrations during lockdown (Mid‐March to early June) and post lockdown periods of 2020 temporarily reduced across the four main Canadian cities. The PM_2.5_ concentrations in 2020 are reduced by up to almost 60% across Canada relative to the reference period (2000–2019). The largest anomalies are detected in different months for various cities and may not coincide exactly with the month with the stricter lockdown period due to the superimposed variability associated with weather patterns. However, precipitation and wind speed, that are the two key variables in determining wet and dry deposition and affecting the air pollution and CUHI, did not show any significant anomaly during 2020, with the exception of March in Toronto and May in Calgary. Therefore, we can conclude that the remarkable reduction in PM_2.5_ level with the peak in May and September for Eastern Canadian cities (Toronto and Montreal) and in July for western Canadian cities (Vancouver and Calgary) are due to COVID‐19 lockdowns. In particular, the overall emission reduction of PM_2.5_ is closely linked to a decrease in the vehicle kilometres traveled between the years 2019 and 2020 (APEI, 2020; Tian, An, et al., 2021; Tian, Cui, et al., 2021). The transportation sector, primarily driven by on‐road vehicle emissions in urban areas, is the second‐largest source of fine particulate matter, accounting for 33% of total PM_2.5_ in Toronto, around 25% in Montreal and Vancouver, and 18% in Calgary. In all four cities, over half of the total PM_2.5_ emissions originate from the industrial sector, with the upstream oil and gas industry being the primary source of PM_2.5_ emissions in Calgary (Mashayekhi et al., 2021).

In general, the results obtained in this study are consistent with previous findings looking at the impact of COVID‐19 on air quality in other countries. For instance, the level of PM_2.5_ decreased during COVID‐19 period in 2020 relative to the reference period (2017–2019) in the continental United States (Berman & Ebisu, 2020). Berman and Ebisu (2020) highlighted that this decrease is statistically significant in urban counties from states that implemented early non‐essential business closures. Ground‐level observations around California indicate a 31% drop in the concentration of PM_2.5_ during the lockdown (March–May) compared to the two and half months preceding the lockdown (January–March) in 2020. The decrease in PM_2.5_ levels is 19% greater than the average of the preceding 5 years (2015–2019) for the same period (Liu et al., 2021). Parker et al. (2020) showed that the 2020 concentration of PM_2.5_ across the South Coast Air Basin in California during the first lockdown showed an overall reduction compared to the same period of the previous 5 years due to an up to 50% decrease in traffic up to 50%. Finally, Son et al. (2020) show that the concentration of PM_2.5_ during the mitigation period (30 days after the emergency declaration was enacted by each state) decreased relative to baseline period (30 days before the mitigation period) for most states in the U.S. ranging from 0.25 μg/m^3^ (4.3%) in Maryland to 4.20 μg/m^3^ (45.1%) in California mainly because of notable decreases in human activities. In Korea in March 2020, immediately after the start of restrictions, the average level of PM_2.5_ nationwide decreased by ∼17 μg/m3 compared to the mean level of the previous year (45.5%) (Ju et al., 2021).

However, the majority of prior studies have considered a very short reference period (1–5 years) and mostly did not investigate whether particular meteorological patterns may had impacts the PM_2.5_ concentrations. Such short intervals do not allow one to rule out the possibility that the reference period was biased toward a particular extreme. On the other hand, a longer timeseries yields a narrower confidence interval with a smaller margin of error (Figure S20 and Table S11 in Supporting Information S1), which is the variability that arises due to random sampling. As the sample size increases, the estimates become more precise and closer to the true population values (Figure S20 in Supporting Information S1). Therefore, a larger data set has a higher chance of including a diverse range of observations, providing a more accurate representation of the population's characteristics. Our study is the first that uses long‐term ground‐based monitoring data (20 years) to obtain a reliable comparison of changes in pollutant concentrations during COVID‐19.

In this study, we also show a substantial reduction in canopy UHI during lockdown periods, as evidenced by the negative differences in canopy UHI intensity between 2020 and the preceding 20‐year reference period for most populated cities in Canada. The observed reduction in CUHI in the time of the lockdown period is primarily attributed to the decrease in anthropogenic heat release resulting from the closure of road transportation, shutdown of nonessential businesses and industrial activities across Canada which is in line with previous studies. Ali et al. (2021) showed that restrictions on transportation in the cities during the lockdown periods, led to a noticeable decline in the surface UHI effects in megacities across Pakistan (Ali et al., 2021). During the lockdown period of 2020, the average level of the surface UHI declined by 19.2% over the United Arab Emirates (Alqasemi et al., 2021) and by 59.8% over Spain (Hidalgo García & Arco Díaz, 2022) compared to the same period in 2019. The surface UHI and canopy UHI intensities across numerous megacities in China are reduced by 25% and 36%, respectively, during daytime and declined by 20% and 42%, respectively, at night in the lockdown periods of 2020 relative to the same period of 2017–2019. These reductions are primarily attributed to the significant decline in anthropogenic activities resulting from stringent lockdown measures (Liu et al., 2022). As a result of the May 2020 lockdown, the surface UHI reduced from 22.5% to 19.4% in Dhaka city in Bangladesh due to significant decrease in human activities (Sresto et al., 2022).

Wind speed, cloudiness and rainfall are crucial meteorological parameters in terms of their impact on UHI intensity, as they affect long wave radiation losses and short wave absorption, and vertical and horizontal atmospheric mixing (Al‐Obaidi et al., 2021). Our findings show that meteorology could not be the dominant driver of substantial decline in canopy UHI during lockdown and post lockdown of 2020 across the four Canadian cities analyzed here.

We acknowledge that our study's scope was limited to four major Canadian cities with available long‐term data. This narrows the applicability of our conclusions to a specific subset of urban centers, preventing broader generalizations about the impacts of COVID‐19 lockdowns on urban climates in the entire country. Additionally, relying on single measurements for entire cities restricts the comprehensiveness and precision of our findings, potentially overlooking localized variations in temperature and air quality. Moreover, in quantifying the UHI effect, the accuracy of considering certain rural stations might be compromised due to the influence of distinct microclimates. Nonetheless, given the absence of alternative stations possessing relevant data in close proximity to the downtown station, our choices were unfortunately constrained. As such, future research should endeavor to include more diverse locations with reliable data to enhance the overall understanding of the effects of lockdown measures on urban environments across Canada. Furthermore, inhomogeneous distribution of PM_2.5_ between urban centers and suburban areas contribute to changes in UHI intensity (Crutzen, 2004; Wu et al., 2021). The relationship between UHI and PM_2.5_ concentration is influenced by how aerosols interact with radiation, evaporation, and the height of the planetary boundary layer (Yang et al., 2021, 2022). Although exploring these dynamics was beyond the scope of this study, future research should investigate the direct and indirect effects of PM_2.5_ and the impact of scattering and absorption by PM_2.5_ on UHI intensity.

Lastly, the change in emission patterns due to indoor activities during the COVID‐19 lockdown is an interesting aspect to consider. While investigating this element is outside the scope of the present study, we acknowledge that it could hold promise as an area for future research with the aim of improving air quality in megacities.

## 5 Conclusions

In this study, we present evidence from a city‐wide perspective showing a connection between reduced human emissions and changes in both urban pollution and UHI. Our results provide key insights of the impact of anthropogenic versus natural emissions on urban environment. We emphasize that the influence of meteorological parameters is extremely important and should be taken into account, especially if a short timeframe is used to draw conclusions on the impact of COVID‐19 on CUHI or air pollution. However, prior research has largely ignored this aspect, resulting in the possibility that observed changes in air quality and CUHI intensity shown in other regions during lockdowns may not be solely due to reduced human activities, but instead could be caused by meteorological variations occurring at the same time as the outbreak of COVID‐19.

## Conflict of Interest

The authors declare no conflicts of interest relevant to this study.

## Supporting information

Supporting Information S1

## Data Availability

The air pollutant and meteorological observation data used in this paper can be obtained from Ashraf (2024).
