# Supplementary material for: Impact of Reduced Anthropogenic Emissions Associated With COVID‐19 Lockdown on PM2.5 Concentration and Canopy Urban Heat Island in Canada
Source: Geohealth. 2025 Feb 1;9(2):e2023GH000975. doi: 10.1029/2023GH000975 (PMC11786188; doi:10.1029/2023GH000975)
Supplement: Supplementary file 1 — Supporting Information S1 [file GH2-9-e2023GH000975-s001.docx]

Supporting Information for:

Impact of Reduced Anthropogenic Emissions associated with COVID-19 Lockdown on PM_2.5_ Concentration and Canopy Urban Heat Island in Canada

Samaneh Ashraf ^1,2^, Francesco S.R. Pausata ^2^, Sylvie Leroyer ^3^, Robin Stevens ^1,4^, and Rodrigo Munoz-Alpizar ^5^

^1^ Department of Chemistry, University of Montreal (UdeM), Montreal, QC, Canada

^2^ Centre ESCER (Étude et la Simulation du Climat à l’Échelle Régionale) and GEOTOP (Research Centre in Earth System Dynamics), Department of Earth and Atmospheric Sciences, University of Quebec in Montreal (UQAM), Montreal, QC, Canada

^3^ Meteorological Research Division, Environment and Climate Change Canada, Montreal, QC, Canada

^4^ Climate Research Division, Environment and Climate Change Canada, Victoria, BC, Canada

^5^ Meteorological Service of Canada, Environment and Climate Change Canada, Montreal, QC, Canada

**Corresponding author:**

Samaneh Ashraf ([samaneh.ashraf@umontreal.ca](mailto:ashraf.samaneh@courrier.uqam.ca))

**Author Contributions:**

S.A. and FSR.P. conceived the study; S.A. analyzed the data and wrote the manuscript; S.A., FSR.P. S.L. and R.S. contributed to the interpretation of the results; all authors edited the manuscript.

**Contents of this file:**

Text S1

Figures S1 to S20

Tables S1 to S11

Text S1. Description of the GEOS-Chem Configuration:

We used the very latest version of APEI. Despite its newness, it still only covers 2000-2016 (Crippa et al., 2023). The NEI data is from 2016 and is scaled to other years, with scaling factors spanning 2002-2020. The CEDS emissions data span 1750-2019, according to HEMCO_Config.rc. The AEIC data are for 2019, with scaling factors for 1990-2019. Several natural emissions of aerosols and aerosol precursors are dependent on meteorology, as described in (Keller et al., 2014). These include biogenic VOCs, calculated using the Model of Emissions of Gases and Aerosols from Nature (MEGAN) biogenic emissions module (Guenther et al., 2012); sea spray emissions (Jaeglé et al., 2011); soil and fertilizer NOx (Hudman et al., 2012); desert dust emissions calculated using the Mineral Dust Entrainment and Deposition (DEAD) parameterization (Zender et al., 2003); and lightning-derived NOx (Murray et al., 2012). Technically, some of these emissions are handled both online and offline. Specifically, several natural emissions are calculated online by HEMCO once and then outputted to be read in as offline emissions for computational efficiency. Since these emissions depend solely on meteorology and not on the chemical state of the atmosphere, they remain the same for any simulation using the same meteorological data. We used one months-spin up for our simulations. Black carbon, organic carbon, mineral dust, sea-spray, sulphate, nitrate, and ammonium are the components of PM_2.5_ included in our analysis.


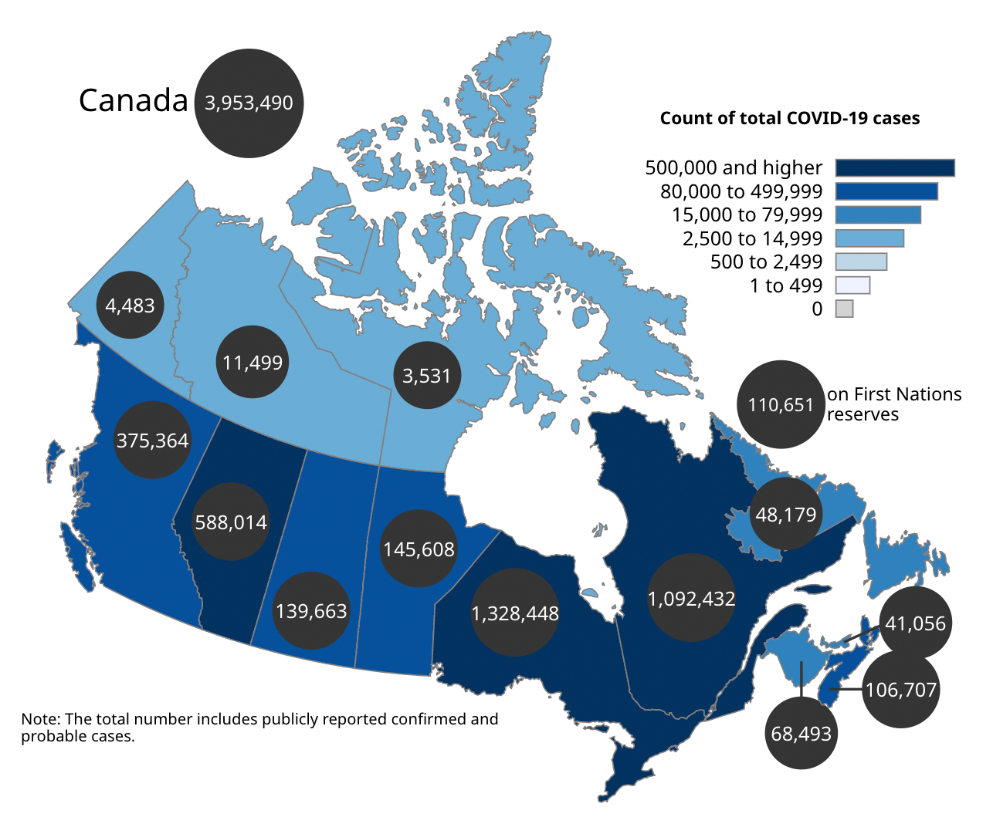


Figure S1. Count of total COVID-19 cases as of July 8, 2022.

Government of Canada, July 8, 2022, 9 am EST. Retrieved July 14, 2022.


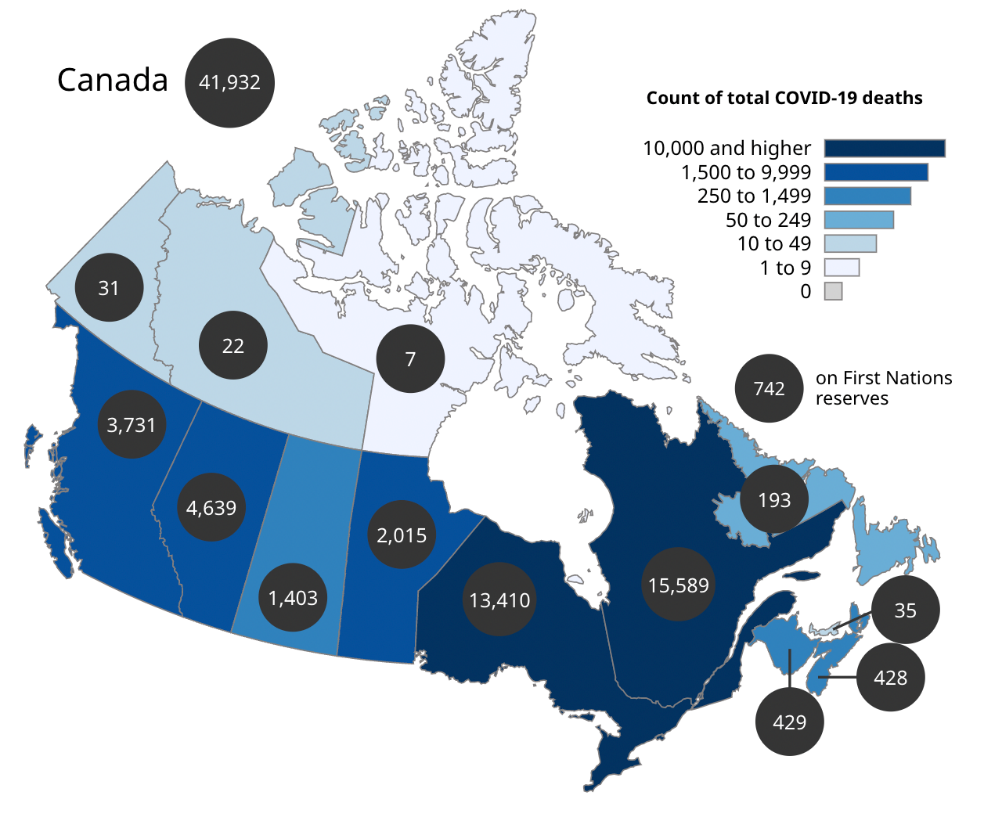


Figure S2. Count of total deaths of COVID-19 cases as of July 8, 2022.

Government of Canada, July 8, 2022, 9 am EST. Retrieved July 14, 2022.


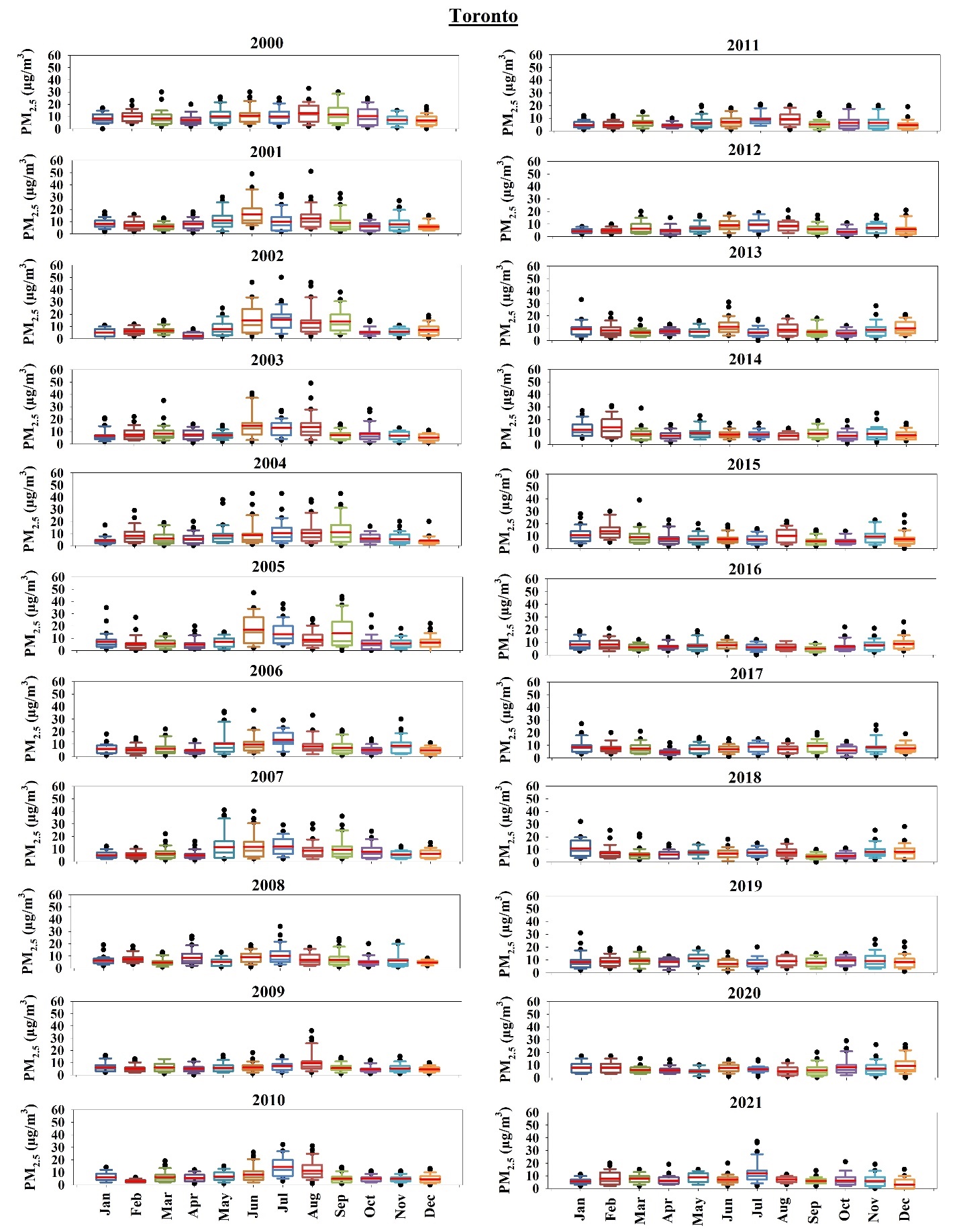


Figure S3. Box plots of observed monthly PM_2.5_ (μg m-3) from 2000-2021 for Toronto.


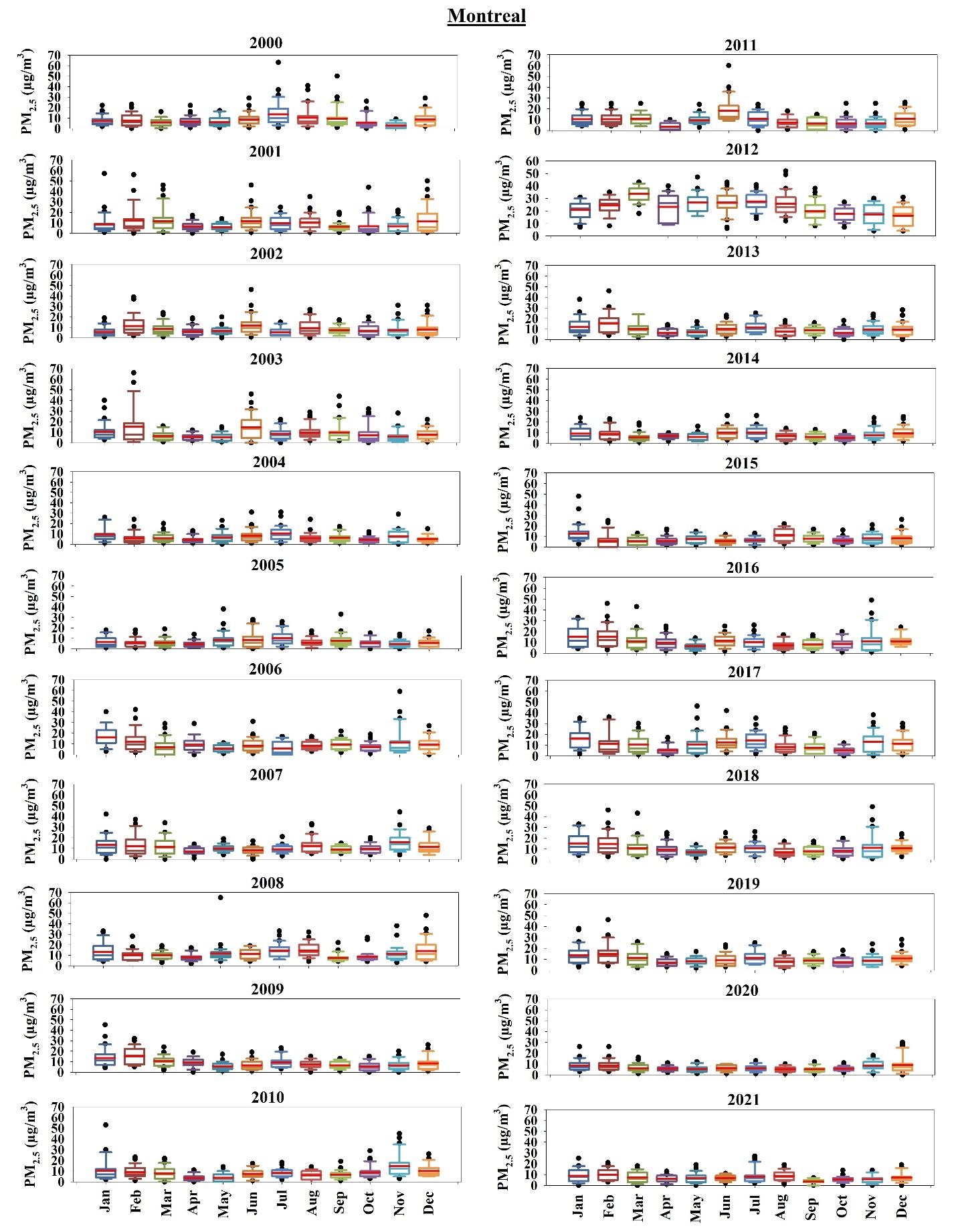


Figure S4. Box plots of observed monthly PM_2.5_ (μg m-3) from 2000-2021 for Montreal.


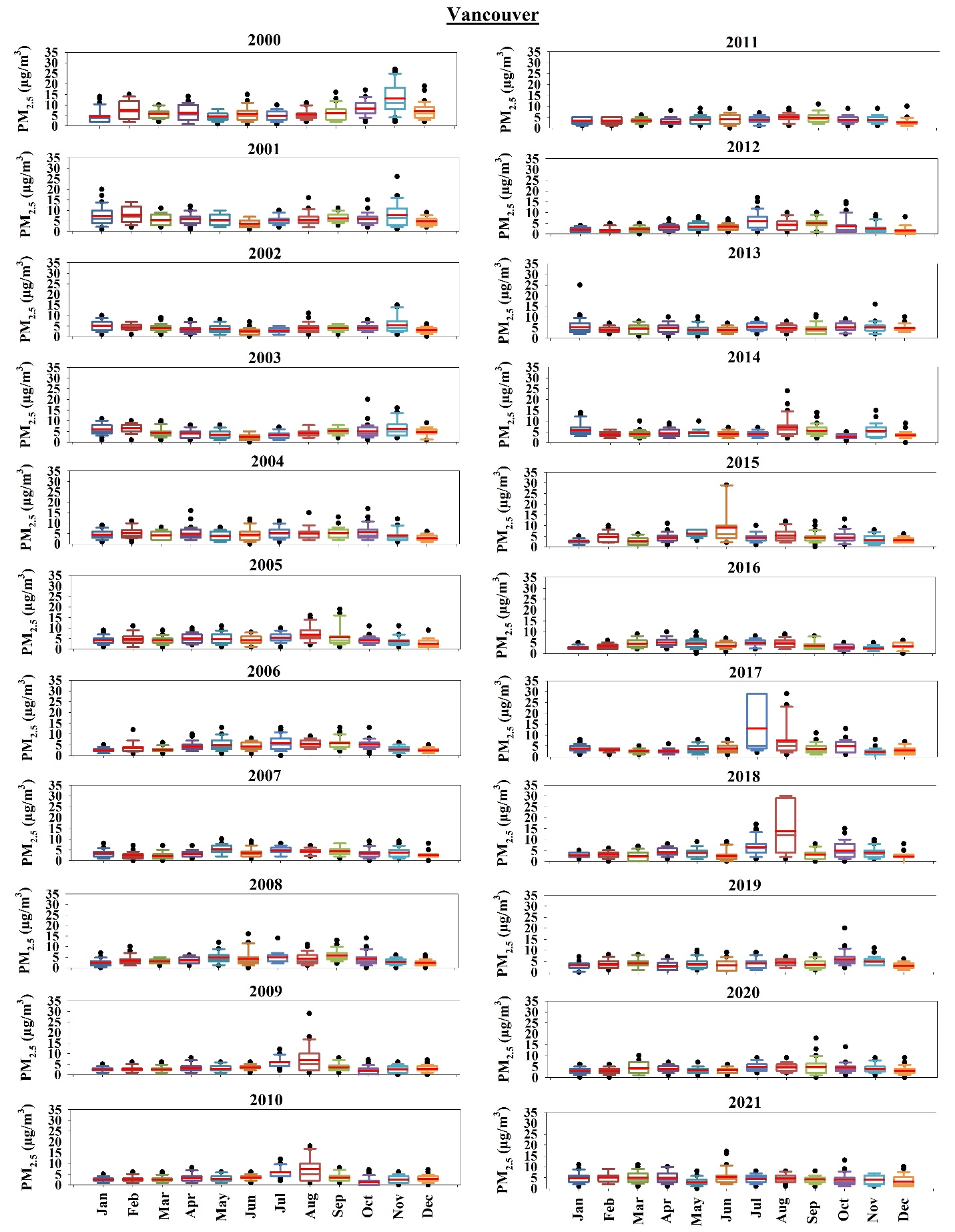


Figure S5. Box plots of observed monthly PM_2.5_ (μg m-3) from 2000-2021 for Vancouver.


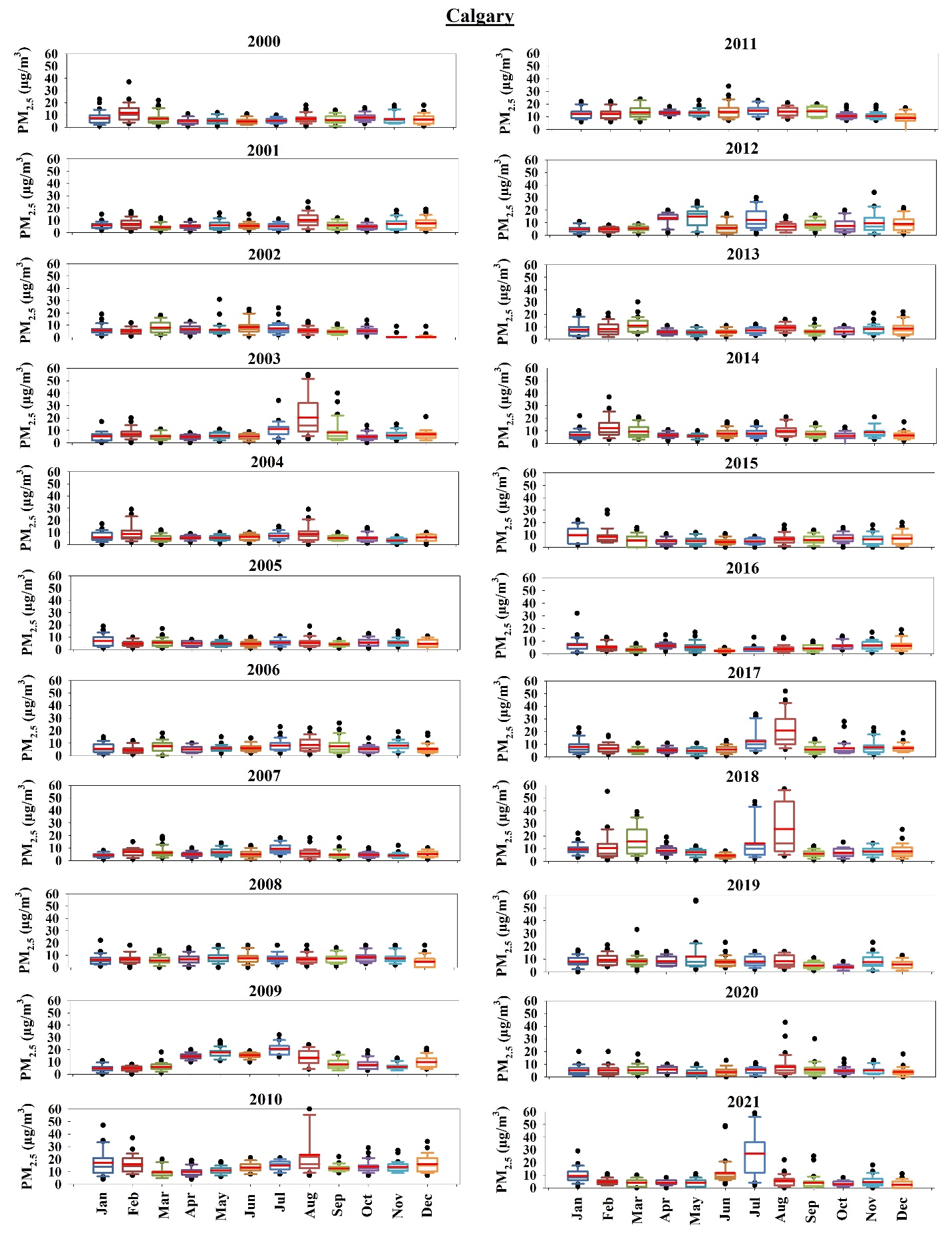


Figure S6. Box plots of observed monthly PM_2.5_ (μg m-3) from 2000-2021 for Calgary.


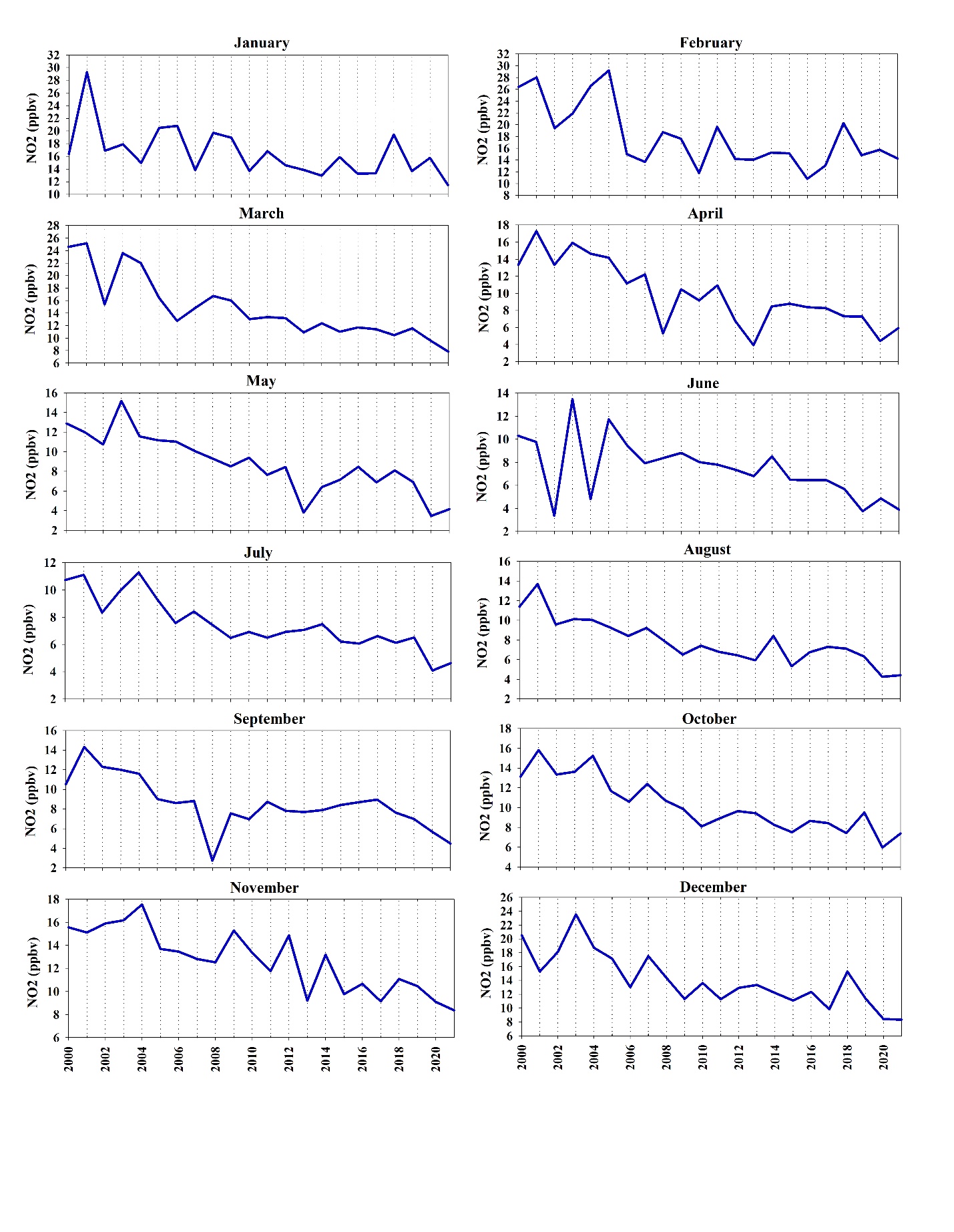


Figure S7. Time series of observed monthly NO_2_ (ppbv) for Montreal downtown from 2000 to 2021.


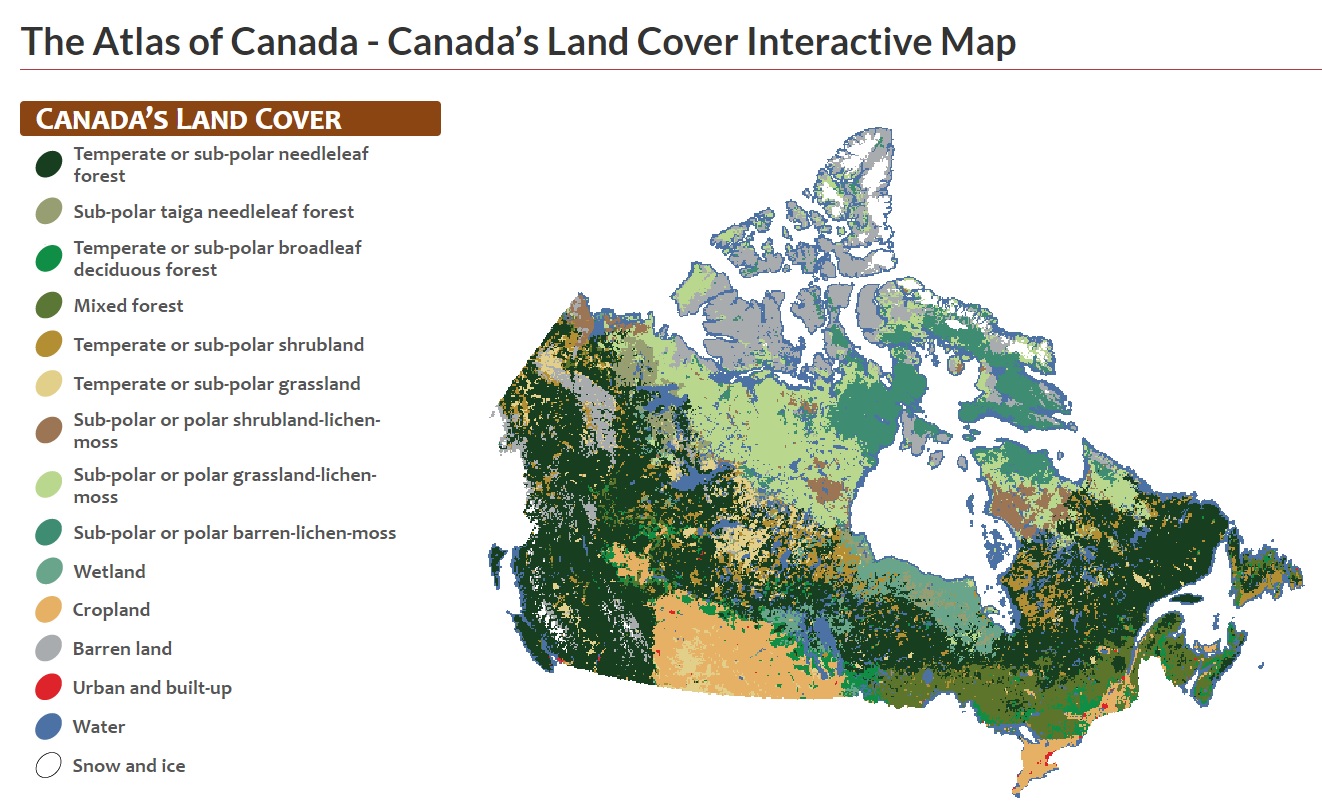


Figure S8. Canada’s land cover interactive map (<https://atlas.gc.ca/lcct/en/index.html>).


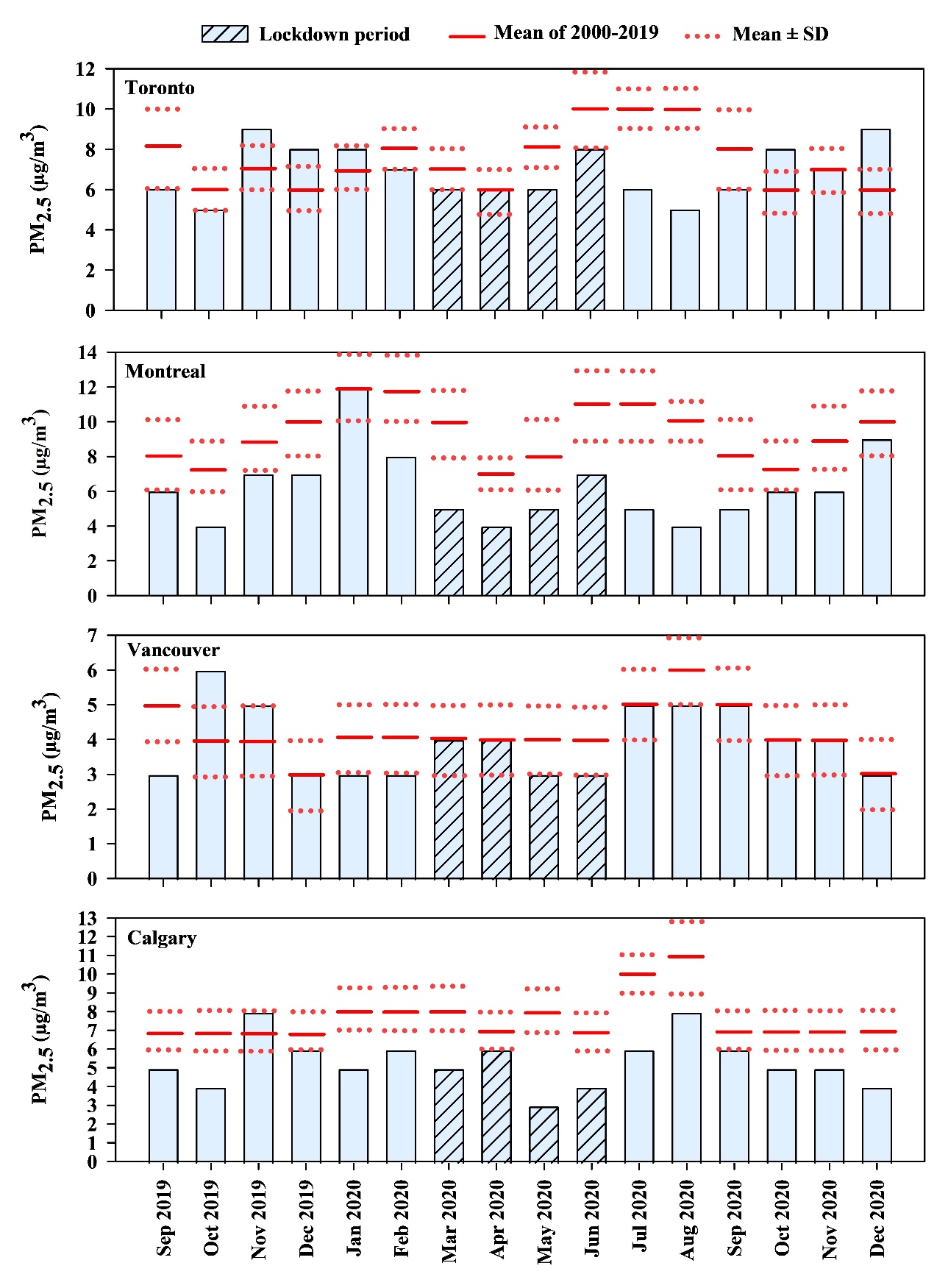


Figure S9. The daily average of PM_2.5_ (μg m-3) from September 2019 to December 2020 relative to the preceding period (2000-2019)


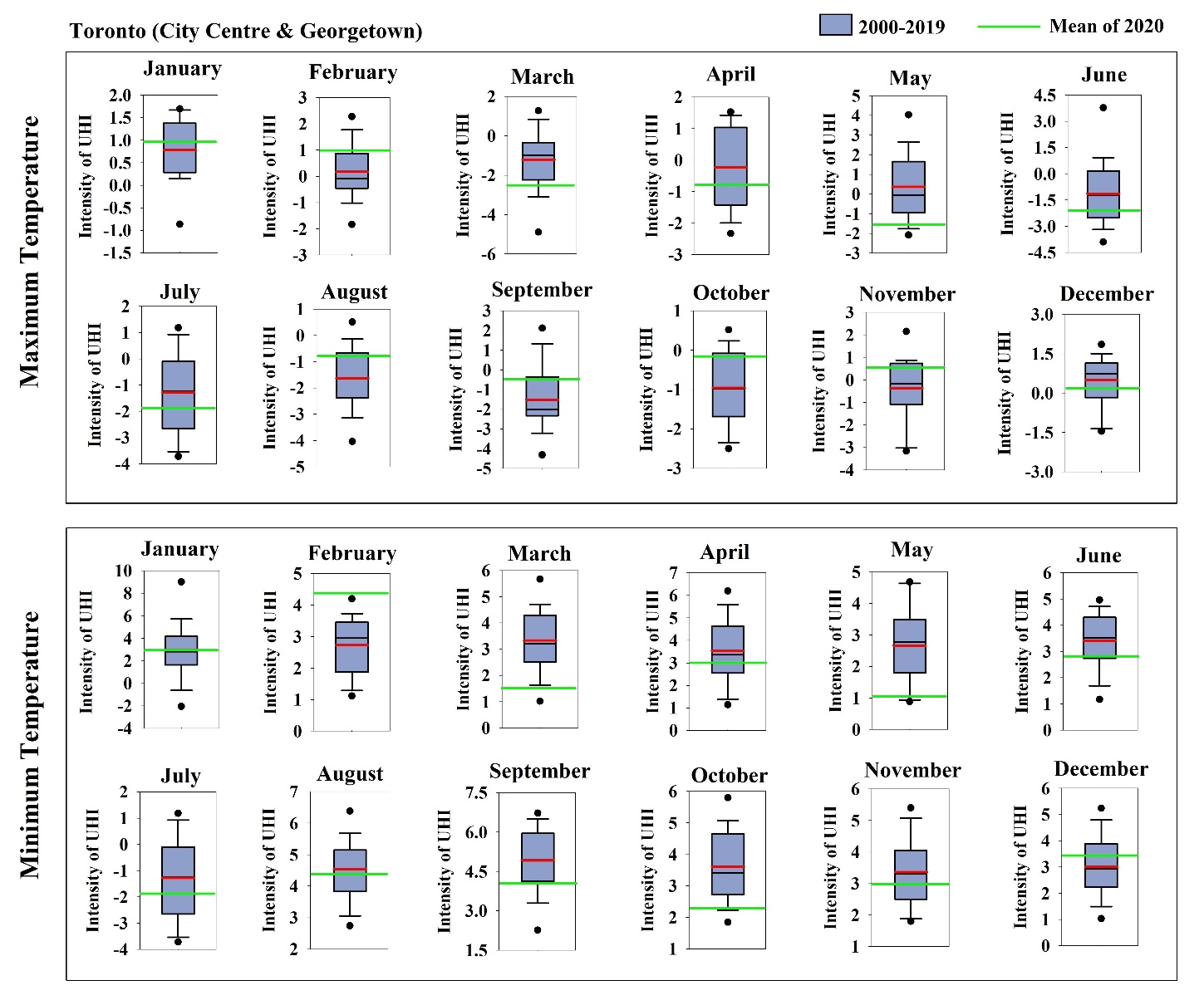


Figure S10. Differences of temperature (intensity of Urban Heat Island) in City Centre (urban area) and Georgetown (rural area) stations of Toronto. The lowest and highest points (the boundary of the lower and upper whisker) are the minimum and the maximum value of the data set. The box is drawn from first quartile to third quartile with a horizontal line drawn in the middle to denote the median. Red line in each box is the mean of the dataset. Black dots are outliers. Green line in each box is the mean of 2020.


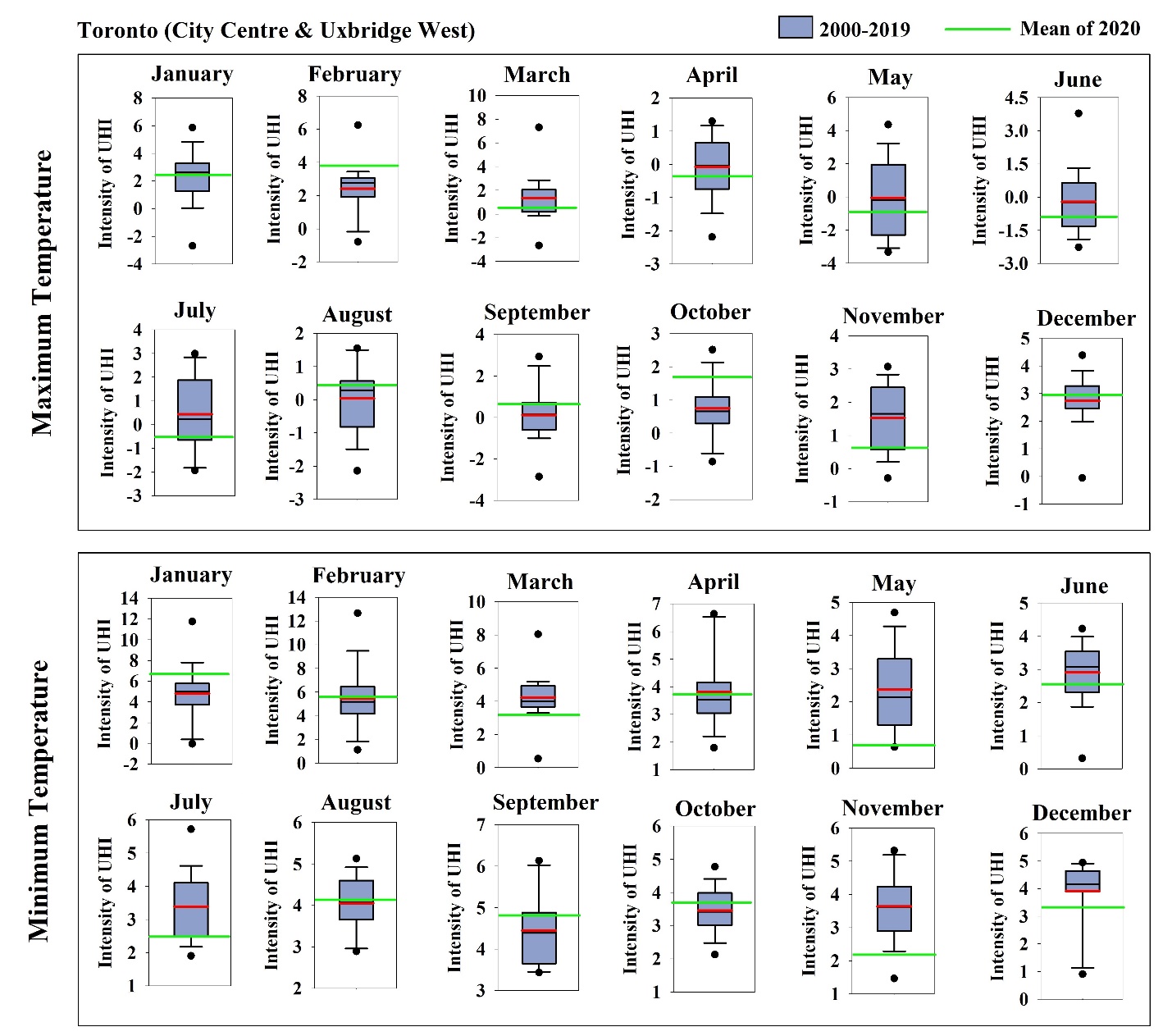


Figure S11. Differences of temperature (intensity of Urban Heat Island) in City Centre (urban area) and Uxbridge West (rural area) stations of Toronto. The lowest and highest points (the boundary of the lower and upper whisker) are the minimum and the maximum value of the data set. The box is drawn from first quartile to third quartile with a horizontal line drawn in the middle to denote the median. Red line in each box is the mean of the dataset. Black dots are outliers. Green line in each box is the mean of 2020.


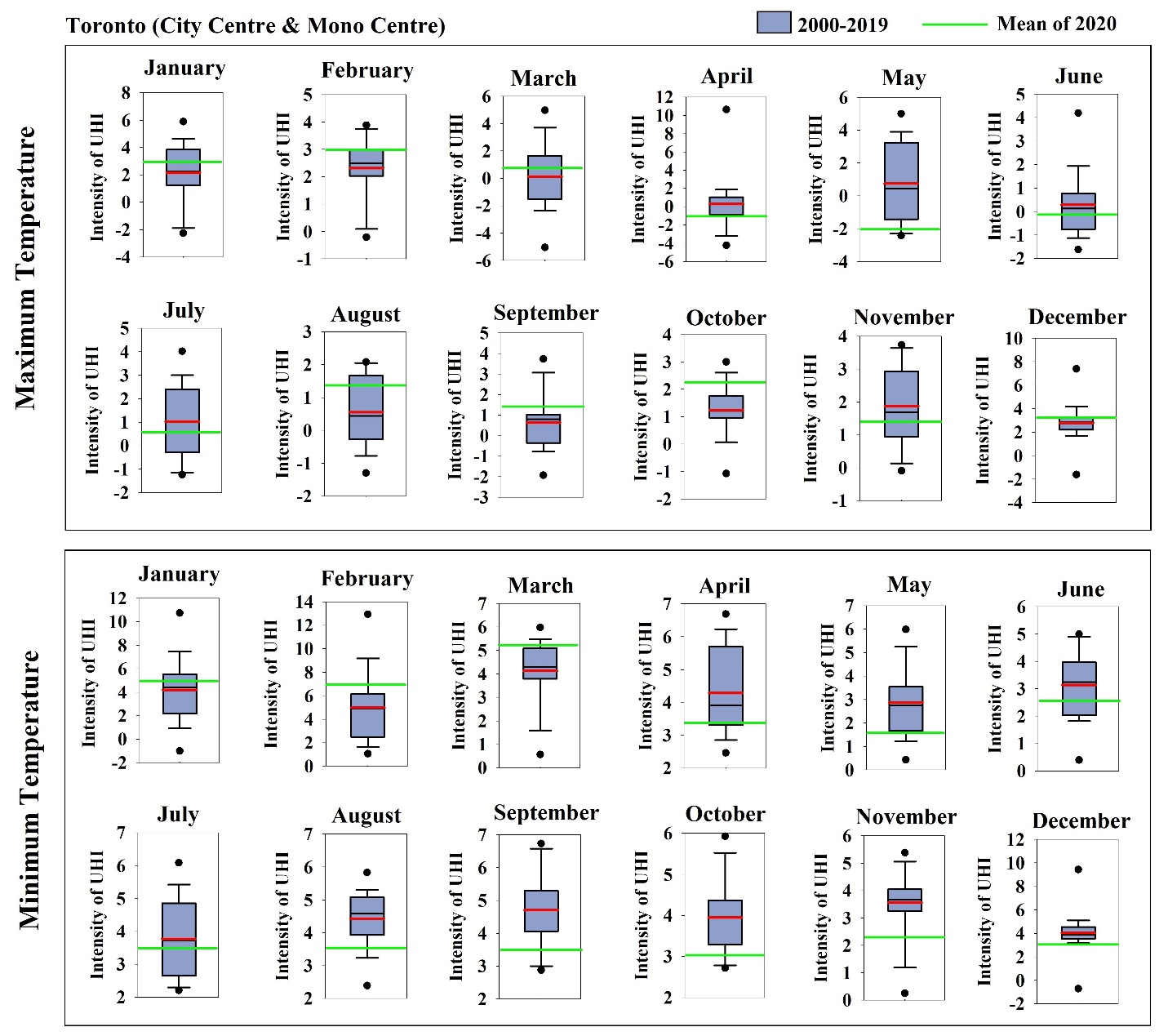


Figure S12. Differences of temperature (intensity of Urban Heat Island) in City Centre (urban area) and Mono Centre (rural area) stations of Toronto. The lowest and highest points (the boundary of the lower and upper whisker) are the minimum and the maximum value of the data set. The box is drawn from first quartile to third quartile with a horizontal line drawn in the middle to denote the median. Red line in each box is the mean of the dataset. Black dots are outliers. Green line in each box is the mean of 2020.


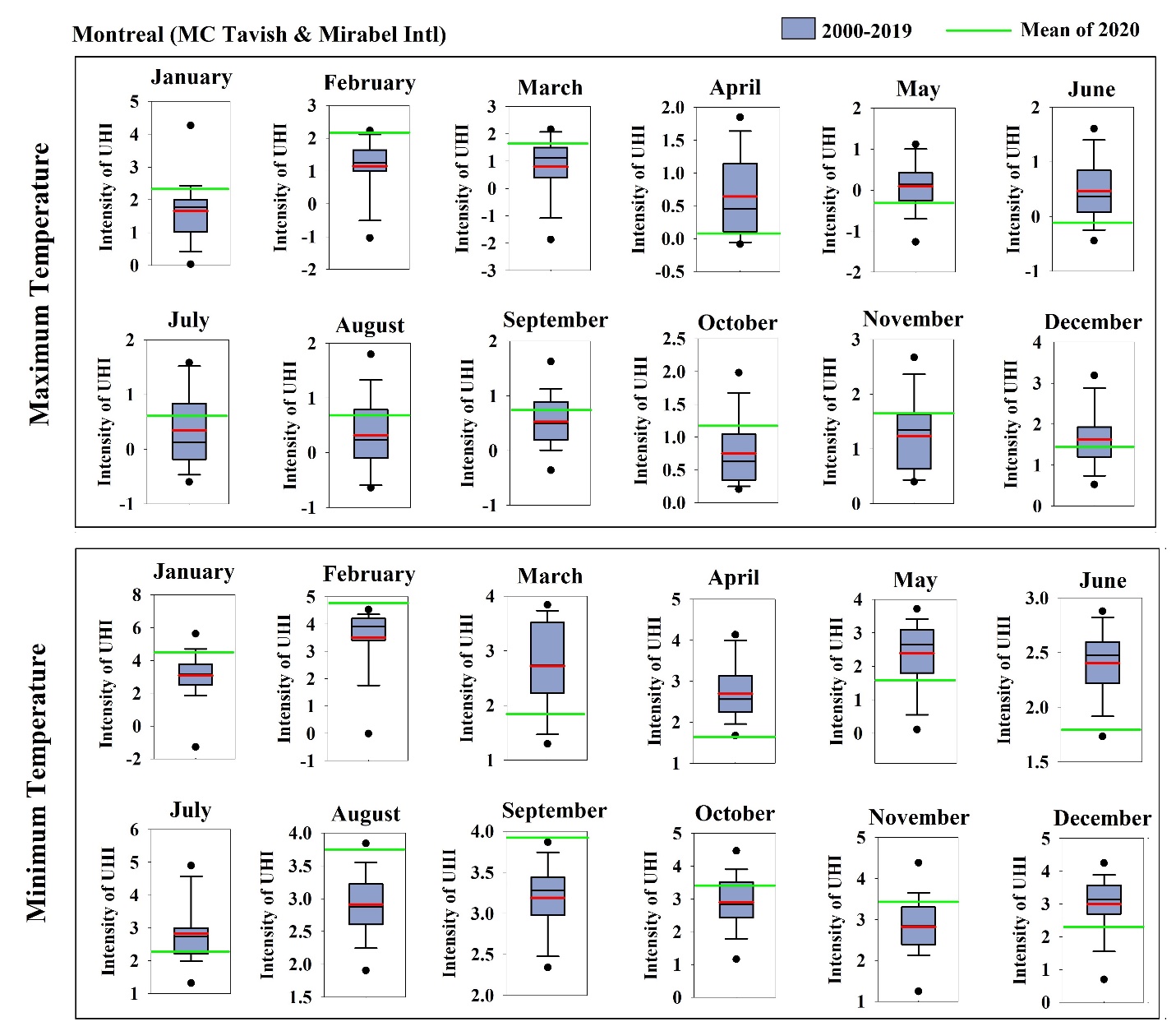


Figure S13. Differences of temperature (intensity of Urban Heat Island) in MC Tavish (urban area) and Mirabel Intl (rural area) stations of Montreal.The lowest and highest points (the boundary of the lower and upper whisker) are the minimum and the maximum value of the data set. The box is drawn from first quartile to third quartile with a horizontal line drawn in the middle to denote the median. Red line in each box is the mean of the dataset. Black dots are outliers. Green line in each box is the mean of 2020.


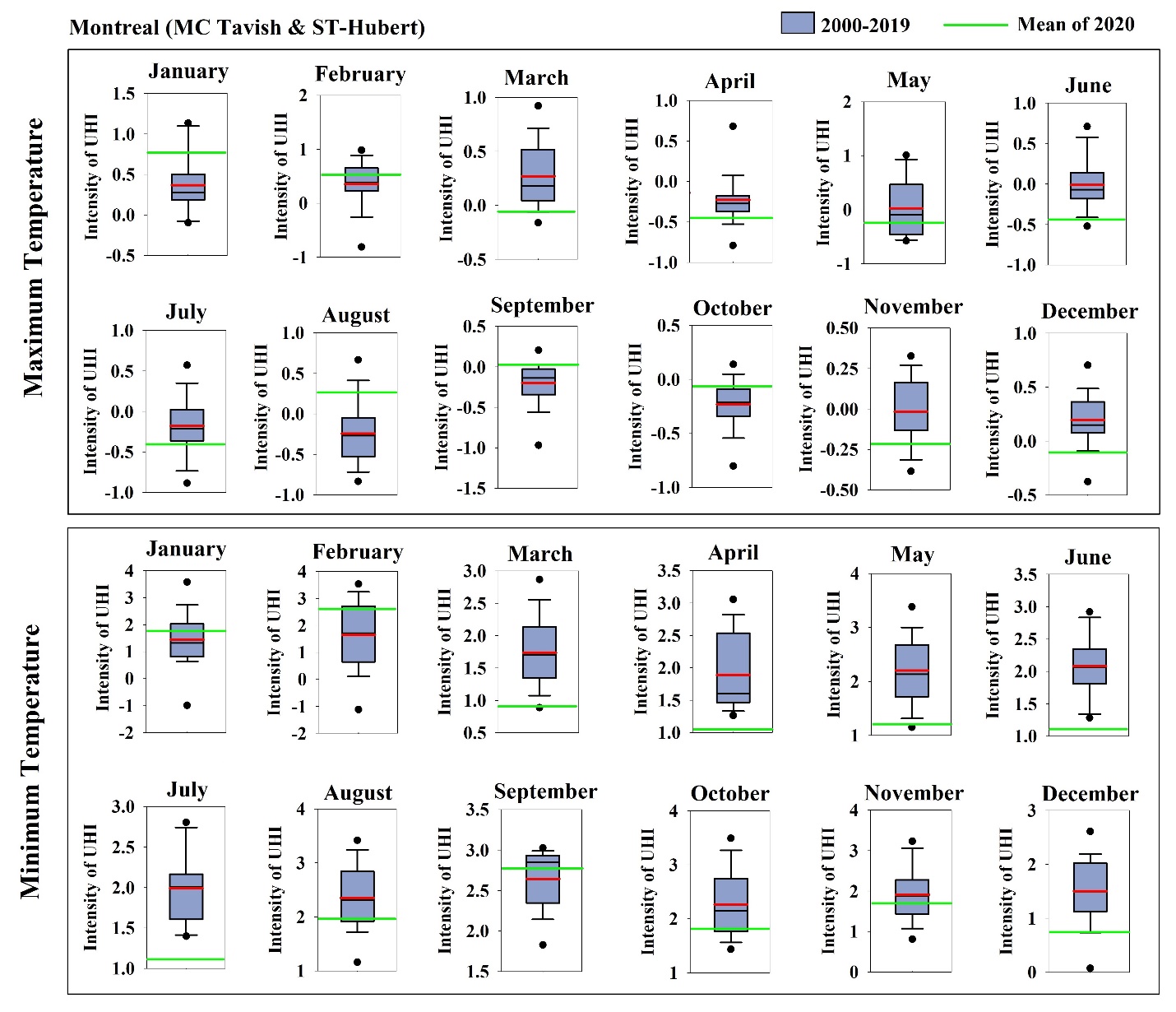


Figure S14. Differences of temperature (intensity of Urban Heat Island) in MC Tavish (urban area) and ST-Hubert (rural area) stations of Montreal. The lowest and highest points (the boundary of the lower and upper whisker) are the minimum and the maximum value of the data set. The box is drawn from first quartile to third quartile with a horizontal line drawn in the middle to denote the median. Red line in each box is the mean of the dataset. Black dots are outliers. Green line in each box is the mean of 2020.


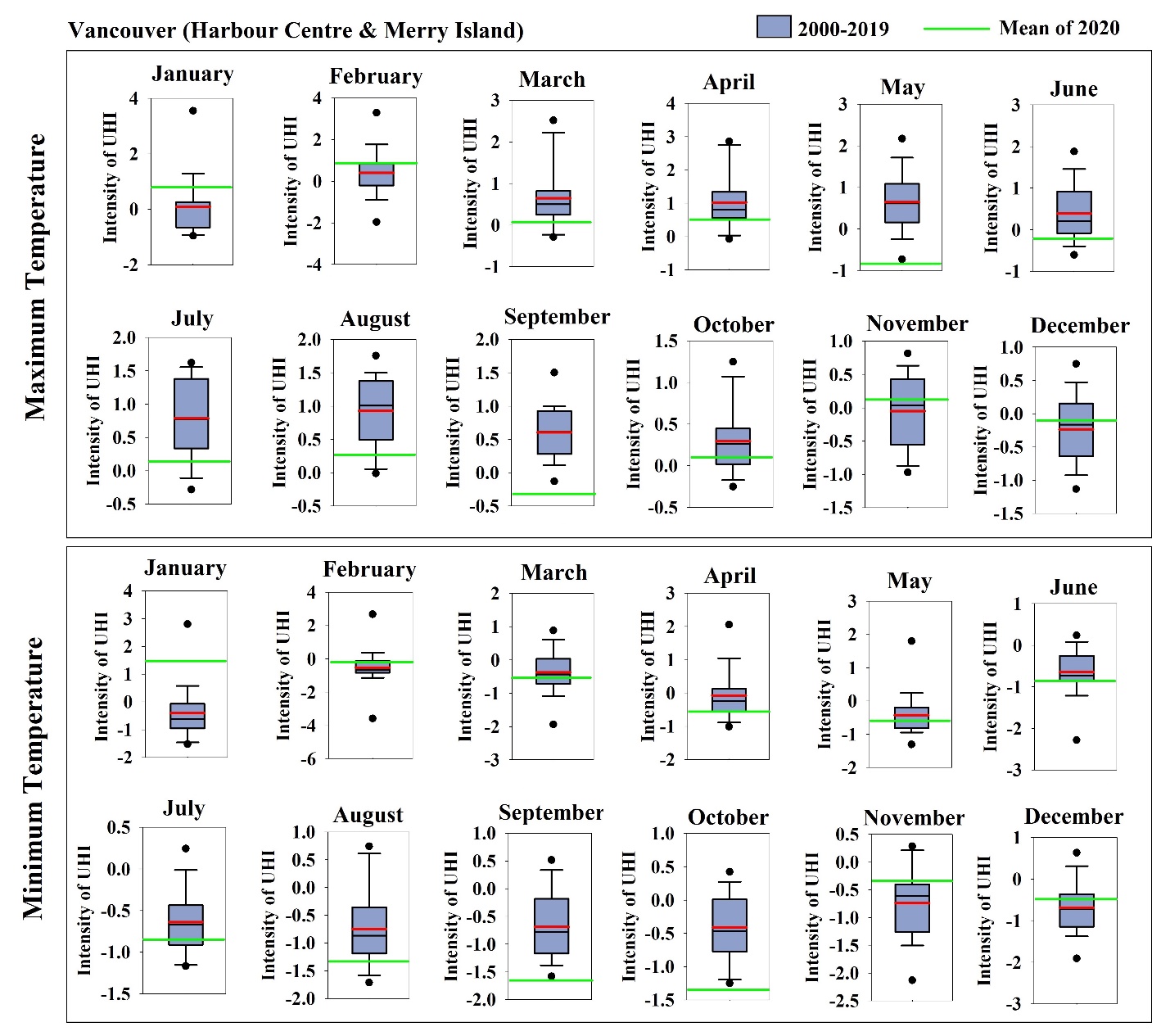


Figure S15. Differences of temperature (intensity of Urban Heat Island) in Harbour Centre (urban area) and Merry Island (rural area) stations of Vancouver. The lowest and highest points (the boundary of the lower and upper whisker) are the minimum and the maximum value of the data set. The box is drawn from first quartile to third quartile with a horizontal line drawn in the middle to denote the median. Red line in each box is the mean of the dataset. Black dots are outliers. Green line in each box is the mean of 2020.


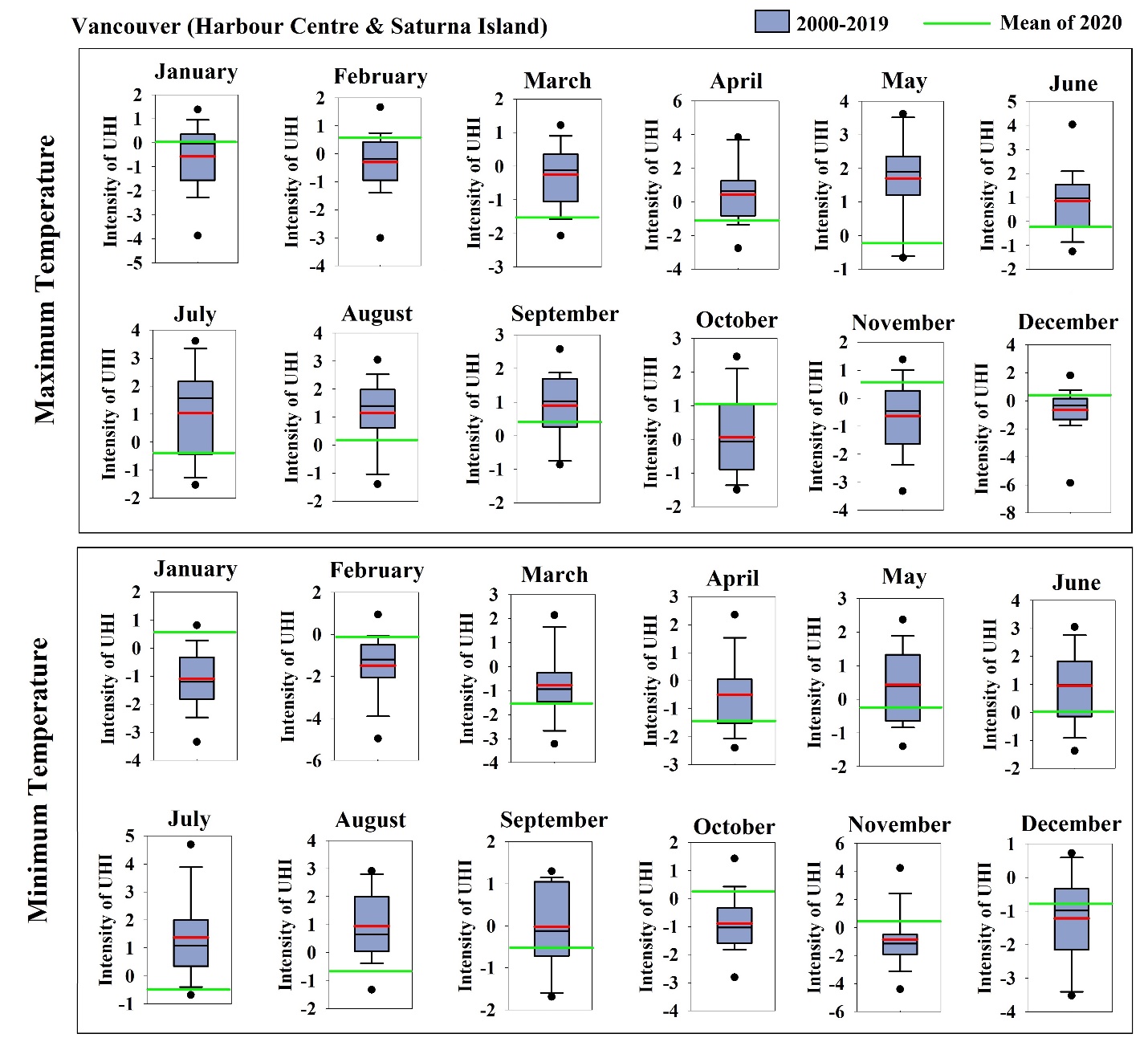


Figure S16. Differences of temperature (intensity of Urban Heat Island) in Harbour Centre (urban area) and Saturna Island (rural area) stations of Vancouver. The lowest and highest points (the boundary of the lower and upper whisker) are the minimum and the maximum value of the data set. The box is drawn from first quartile to third quartile with a horizontal line drawn in the middle to denote the median. Red line in each box is the mean of the dataset. Black dots are outliers. Green line in each box is the mean of 2020.


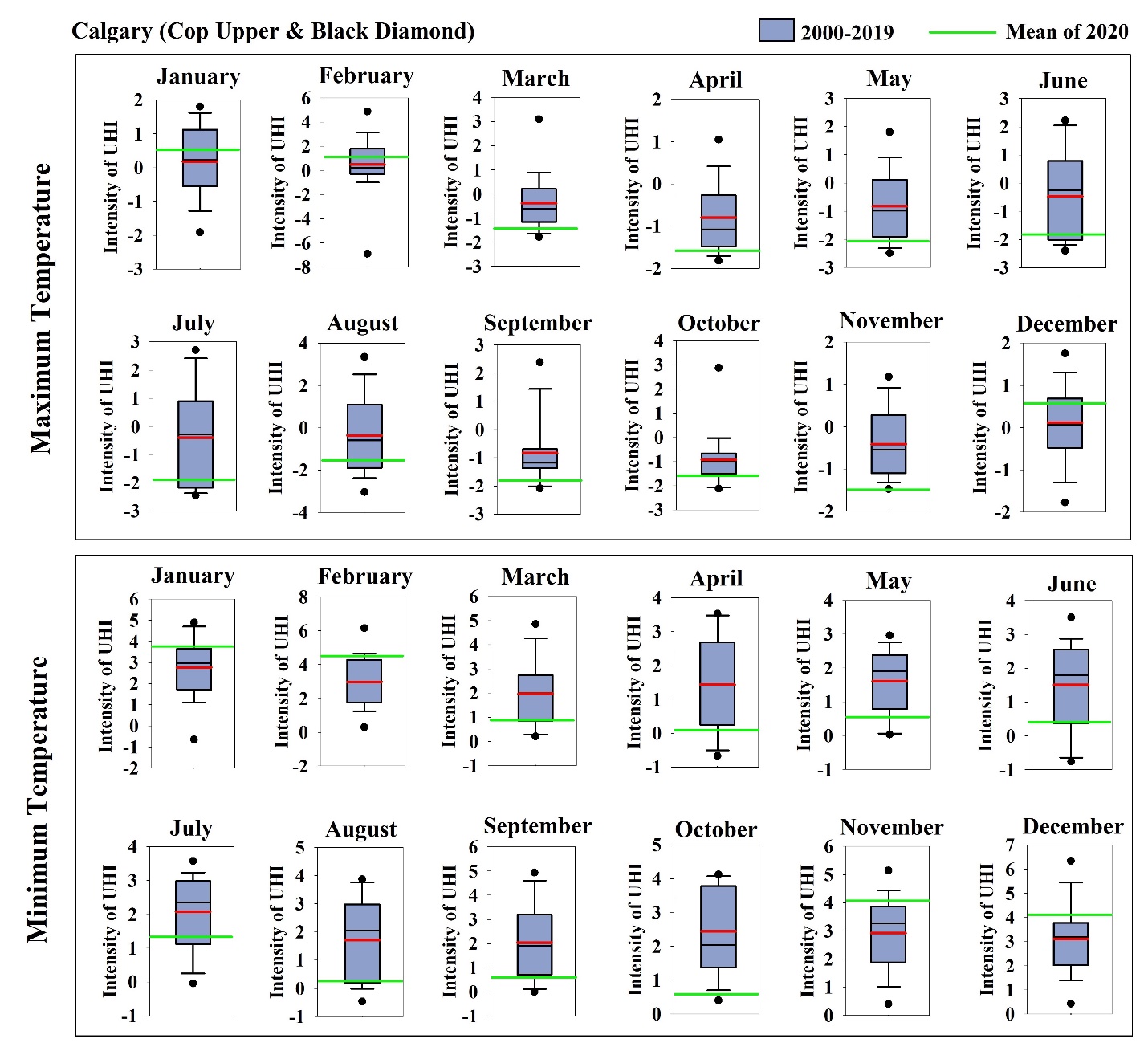


Figure S17. Differences of temperature (intensity of Urban Heat Island) in Cop Upper (urban area) and Black Diamond (rural area) stations of Calgary. The lowest and highest points (the boundary of the lower and upper whisker) are the minimum and the maximum value of the data set. The box is drawn from first quartile to third quartile with a horizontal line drawn in the middle to denote the median. Red line in each box is the mean of the dataset. Black dots are outliers. Green line in each box is the mean of 2020.


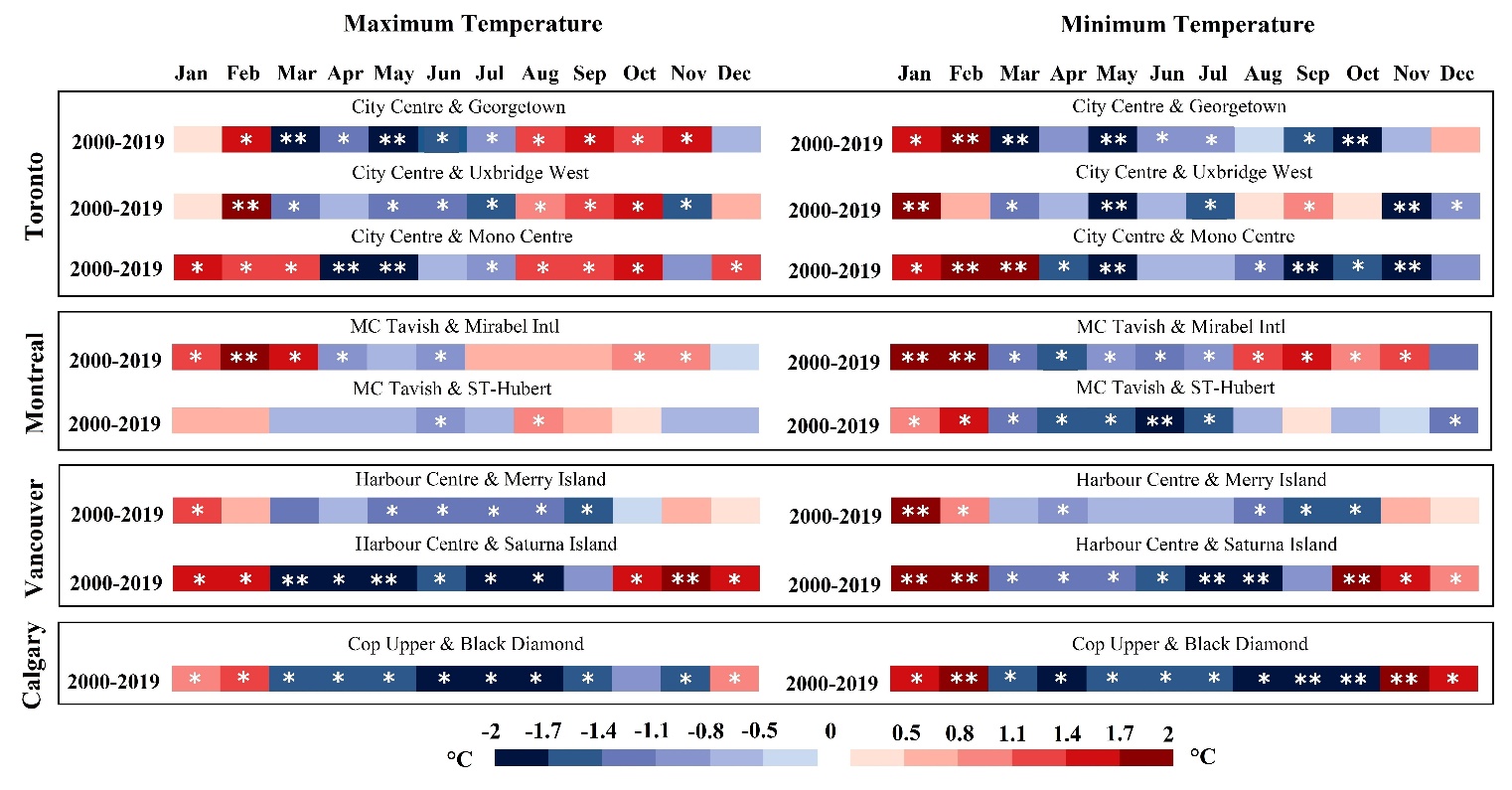


Figure S18. Variations in CUHI intensity in 2020 relative to preceding 20-year-period (2000-2019) based on difference of temperature in urban and rural stations of each city. The asterisks * and ** present observed change is greater than one and two standard deviations of the mean respectively.

**2020s**

**(All emission of 2010-2019)**

**2020s**

**(Anthropogenic emission of 2010-2019)**

**2010-2019**

| 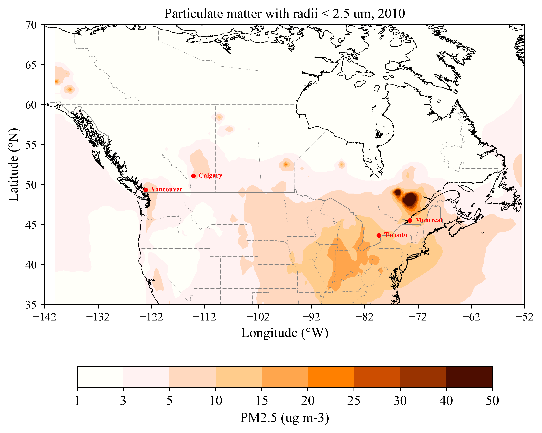 | 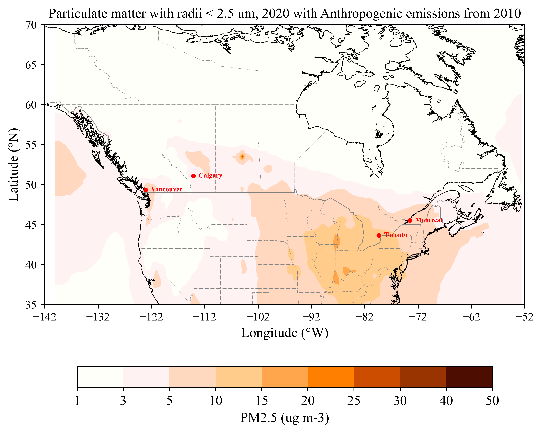 | 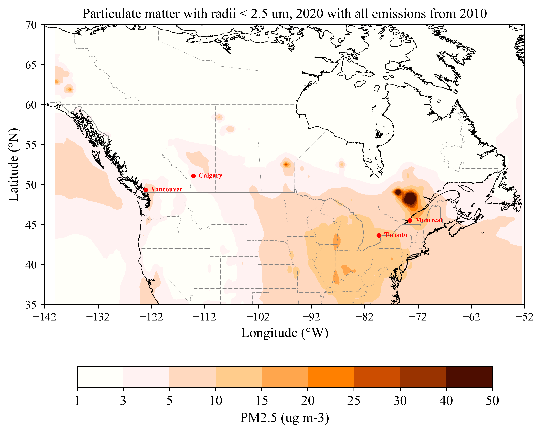 |
| --- | --- | --- |
| 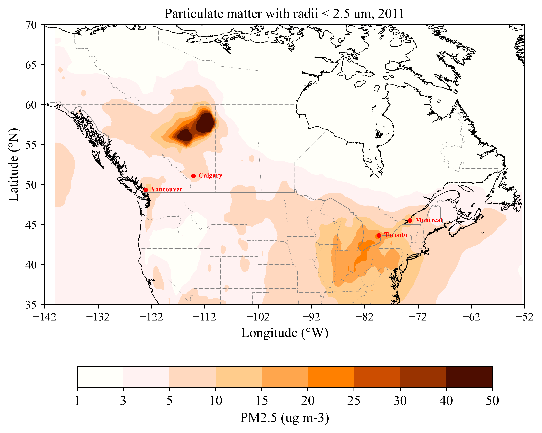 | 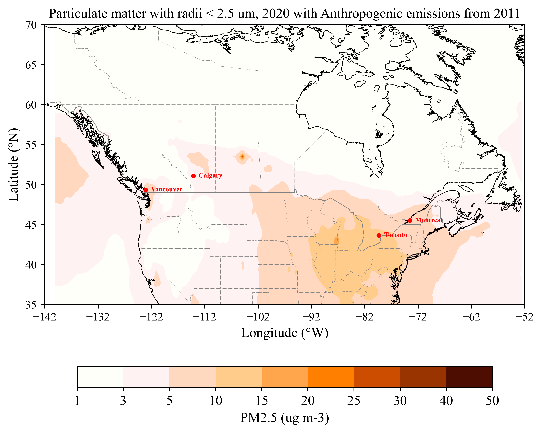 | 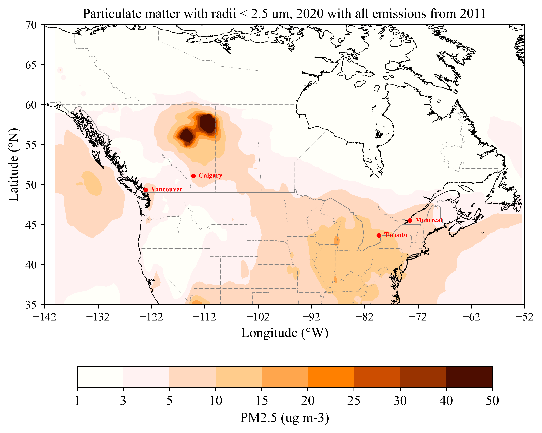 |
| 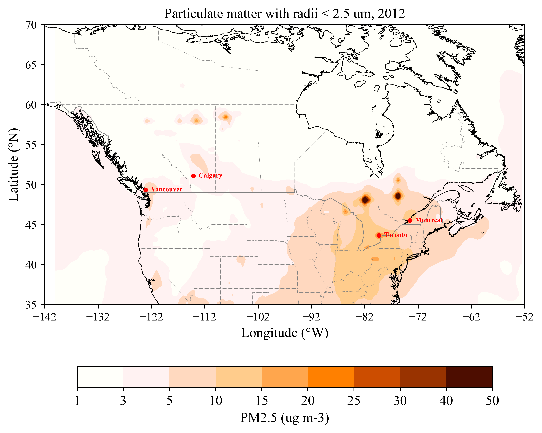 | 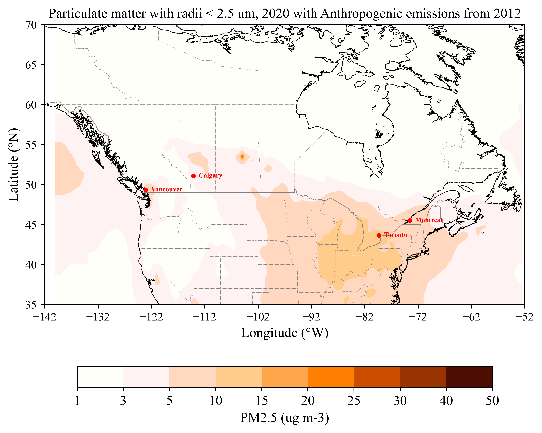 | 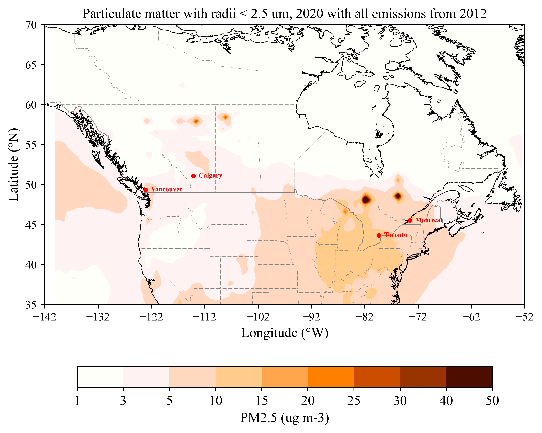 |
| 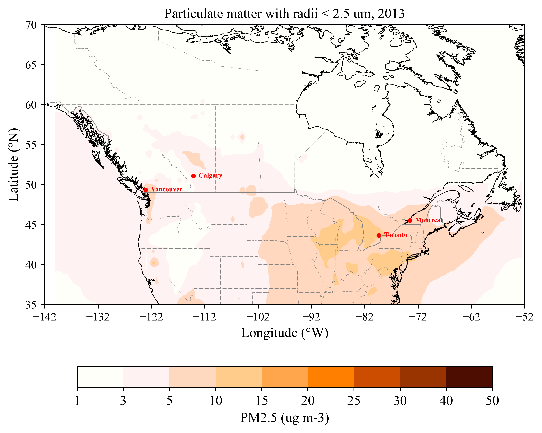 | 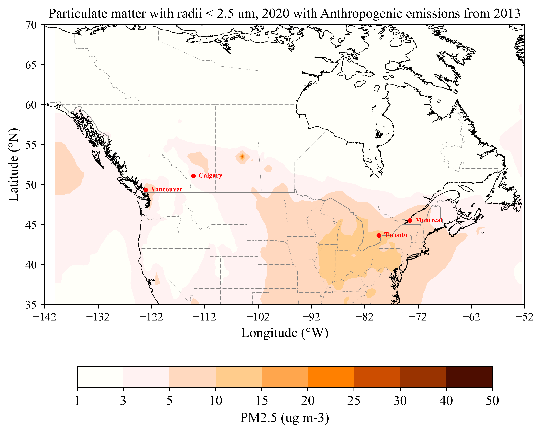 | 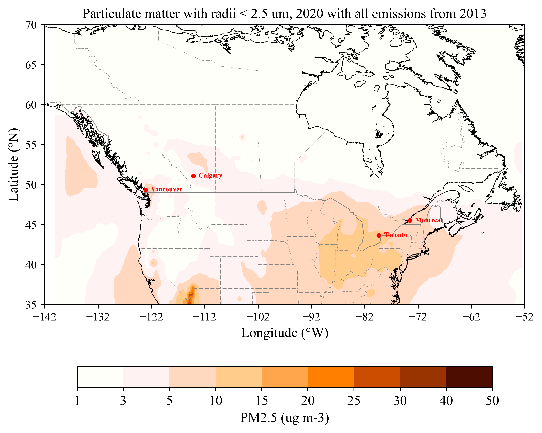 |
| 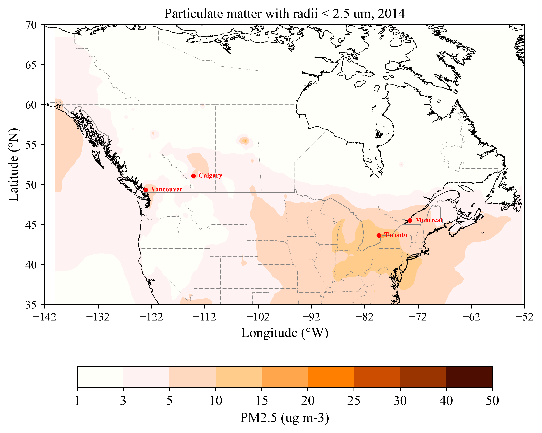 | 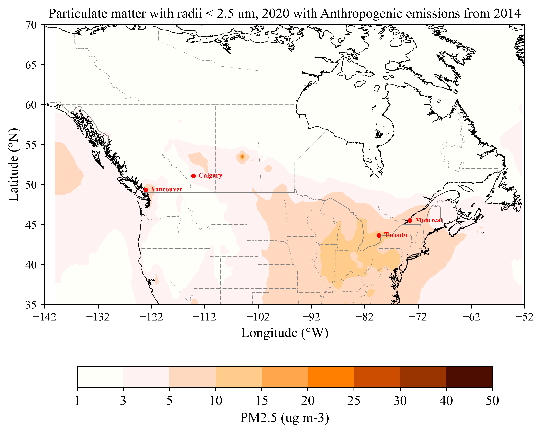 | 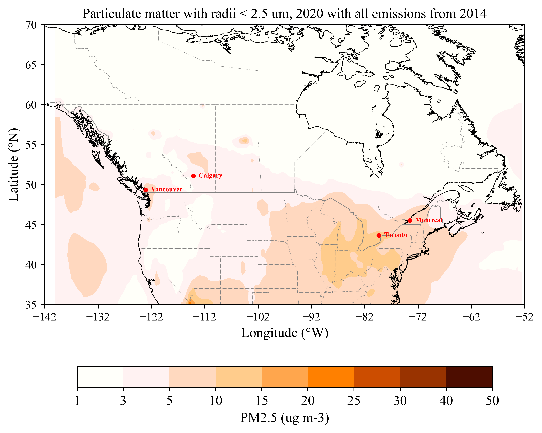 |
| 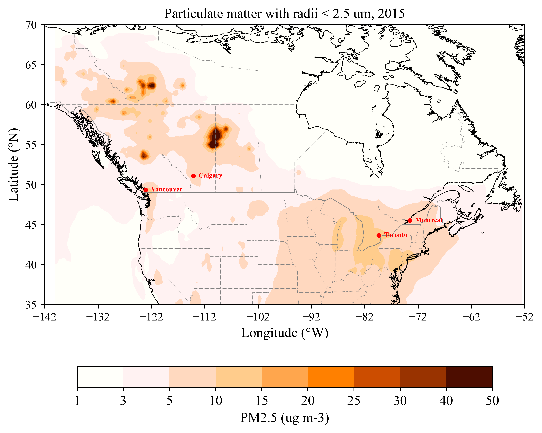 | 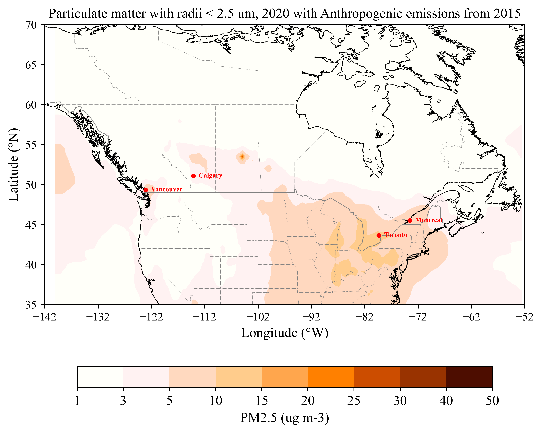 | 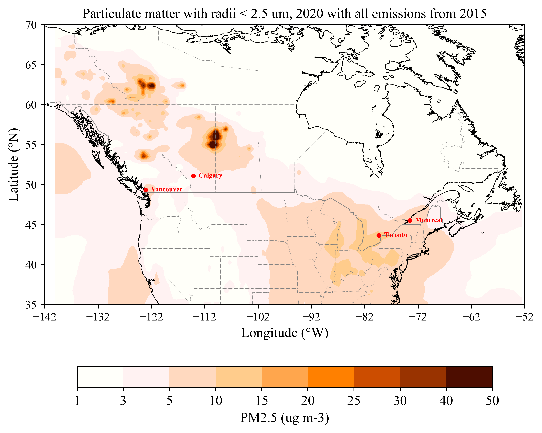 |
| 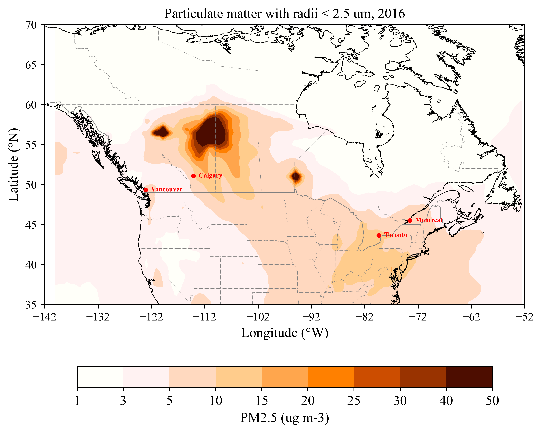 | 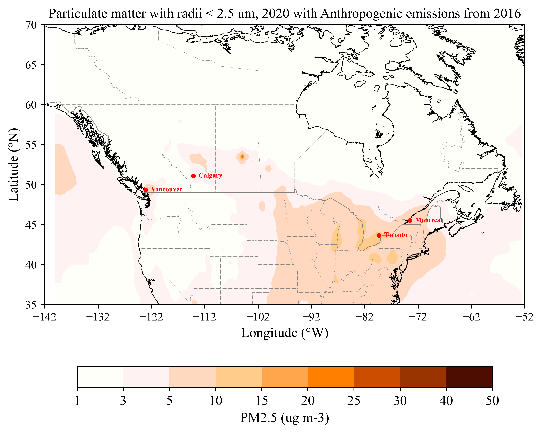 | 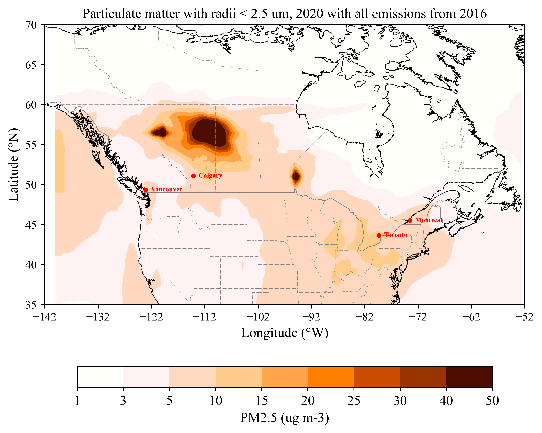 |
| 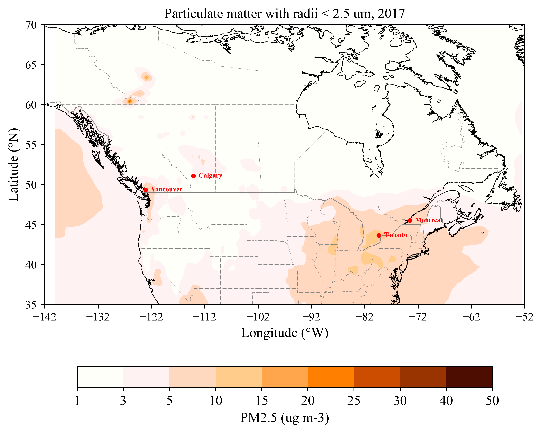 | 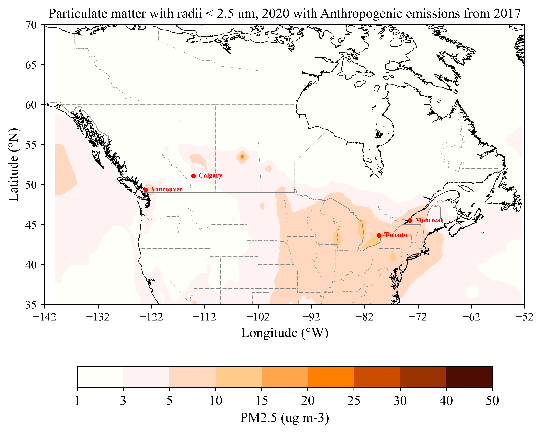 | 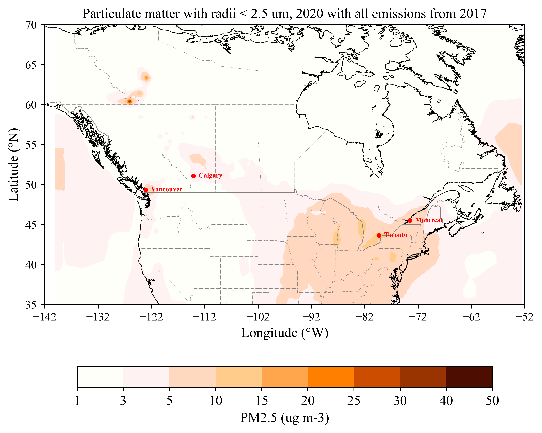 |
| 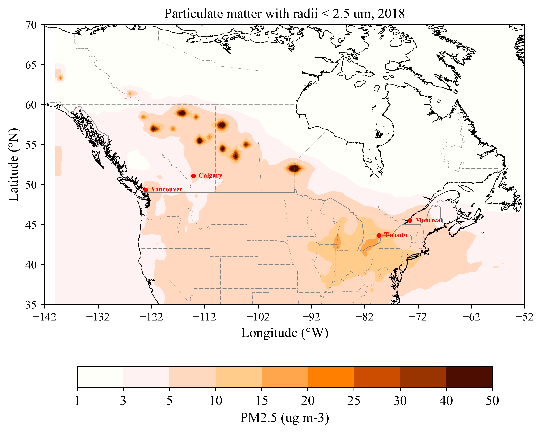 | 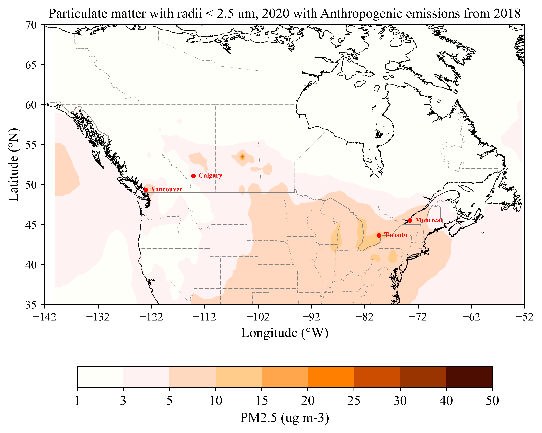 | 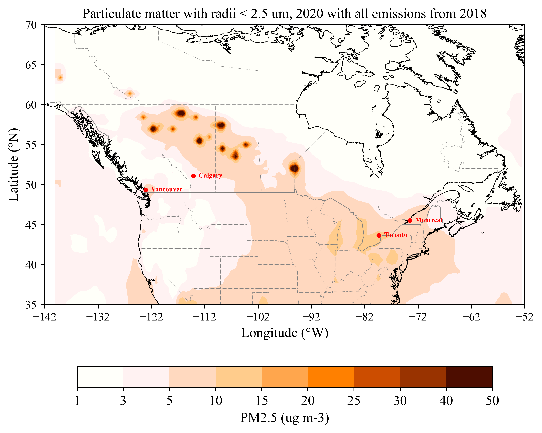 |
| 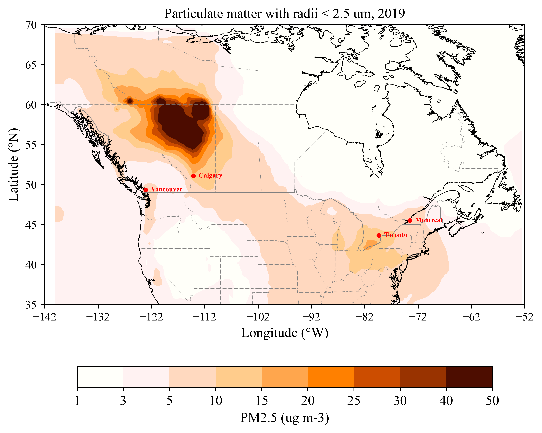 | 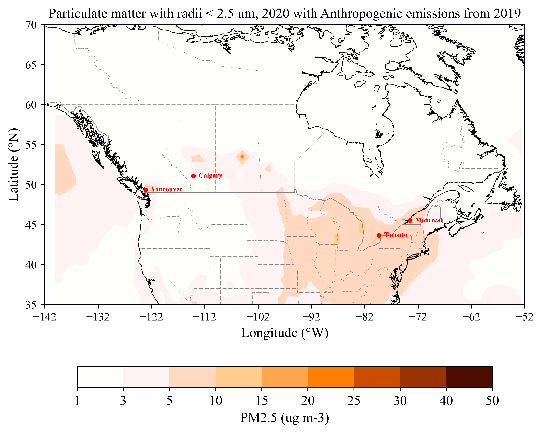 | 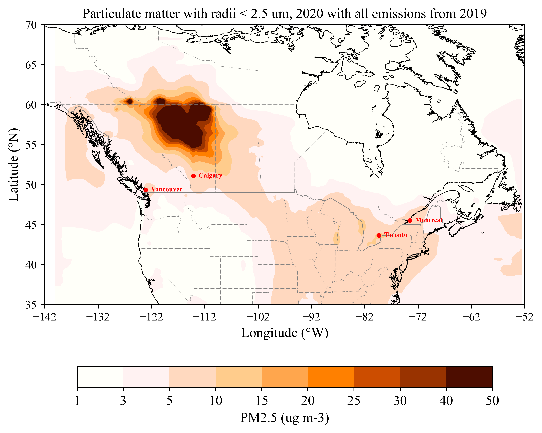 |

Figure S19. GEOS-Chem simulations from 2010 to 2019 (left panel), GEOS-Chem simulation for 2020 using anthropogenic emissions from previous years (2010-2019) (middle panel) and GEOS-Chem simulation for 2020 using all emissions (anthropogenic and natural) from previous years (2010-2019).


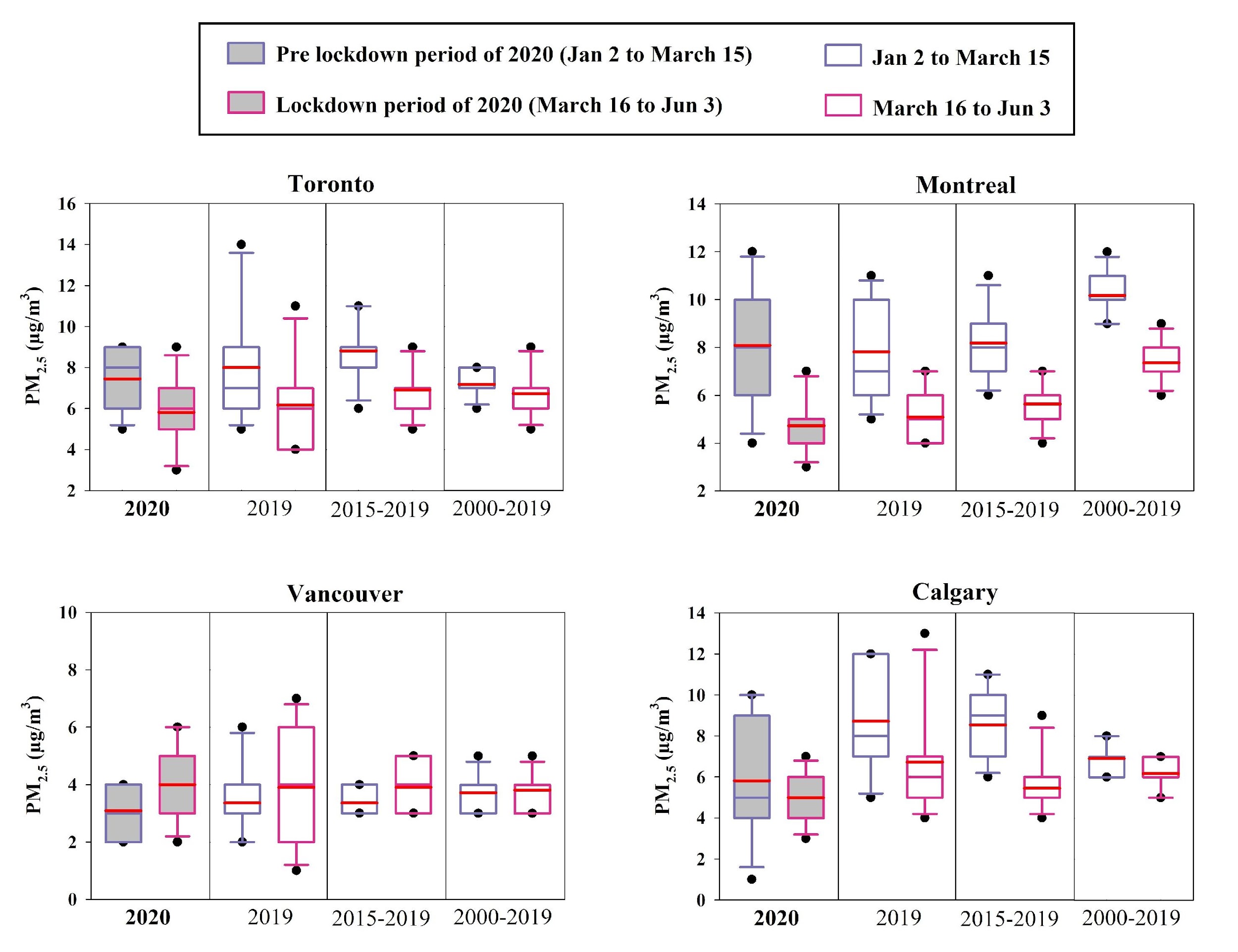


Figure S20. Comparison of daily level of PM_2.5_ (μg/m^-3^) for Toronto, Montreal, Vancouver and Calgary in pre-lockdown and lockdown periods of 2020 using different short and long-term reference periods. The lowest and highest points (the boundary of the lower and upper whisker) are the minimum and the maximum value of the data set. The box is drawn from first quartile to third quartile with a horizontal line drawn in the middle to denote the median. Red bold line in each box is the mean of the dataset. Black dots are outliers.

Table S1. The largest cities in Canada by population.

| Rank | City | Province | Population (Metropolitan area) | Population (City) | Land area (km^2^) | Population density |
| --- | --- | --- | --- | --- | --- | --- |
| 1 | Toronto | Ontario | 6,202,225 | 2,794,356 | 631.10 | 4,427.8/km^2^ |
| 2 | Montreal | Quebec | 4,291,732 | 1,762,949 | 364.74 | 4,833.5/km^2^ |
| 3 | Vancouver | British Colombia | 2,642,825 | 662,248 | 115.18 | 5,749.9/km^2^ |
| 4 | Calgary | Alberta | 1,481,806 | 1,306,784 | 820.62 | 1,592.4/km^2^ |

[Population and dwelling counts: Canada and population centres"](https://www150.statcan.gc.ca/t1/tbl1/en/tv.action?pid=9810001101). [Statistics Canada](https://en.wikipedia.org/wiki/Statistics_Canada). February 9, 2022. Retrieved February 11, 2022.

Table S2. The Geographical characteristics of selected NAPS stations.

| City | Location | Latitude | Longitude | Elevation |
| --- | --- | --- | --- | --- |
| Toronto | Downtown | 43.64 | -79.38 | 85 m |
| Montreal | Downtown | 45.50 | -73.57 | 56 m |
| Vancouver | North | 49.32 | -123.08 | 71 m |
| Calgary | North-West | 51.08 | -114.14 | 1120 m |

Table S3. Mann-Kendall trend analysis of monthly NO_2_, SO_2_, O_3_, and PM_2.5_ for Toronto from 2000 to 2021. The test statistic Z represents the Mann-Kendall Z value, and the asterisk (*) indicates a statistically significant trend at the 95% confidence level.

| **Time series** | **NO_2_** | | **SO_2_** | | **O_3_** | | **PM_2.5_** | |
| --- | --- | --- | --- | --- | --- | --- | --- | --- |
|  | **Test Z** | **Significant** | **Test Z** | **Significant** | **Test Z** | **Significant** | **Test Z** | **Significant** |
| **January** | **-3.11** | ***** | **-2.65** | ***** | **3.11** | ***** | -1.48 |  |
| **February** | **-2.65** | ***** | **-3.58** | ***** | **2.18** | ***** | 0.86 |  |
| **March** | **-2.34** | ***** | **-3.11** | ***** | **2.49** | ***** | 1.25 |  |
| **April** | **-2.65** | ***** | **-2.96** | ***** | **3.11** | ***** | -1.92 |  |
| **May** | **-2.96** | ***** | **-3.27** | ***** | 1.87 |  | 0.93 |  |
| **June** | **-3.89** | ***** | **-3.43** | ***** | -0.78 |  | 0.78 |  |
| **July** | **-2.80** | ***** | **-3.27** | ***** | 0.31 |  | -0.86 |  |
| **August** | **-3.74** | ***** | **-3.11** | ***** | -0.31 |  | -0.62 |  |
| **September** | **-3.27** | ***** | **-3.43** | ***** | -1.09 |  | -0.16 |  |
| **October** | **-3.43** | ***** | **-3.89** | ***** | 0.00 |  | -1.96 |  |
| **November** | **-3.11** | ***** | **-3.43** | ***** | **2.18** | ***** | -0.93 |  |
| **December** | **-2.80** | ***** | **-2.65** | ***** | **3.43** | ***** | 1.96 |  |

Table S4. Mann-Kendall trend analysis of monthly NO_2_, SO_2_, O_3_, and PM_2.5_ for Montreal from 2000 to 2021. The test statistic Z represents the Mann-Kendall Z value, and the asterisk (*) indicates a statistically significant trend at the 95% confidence level.

| **Time series** | **NO_2_** | | **SO_2_** | | **O_3_** | | **PM_2.5_** | |
| --- | --- | --- | --- | --- | --- | --- | --- | --- |
|  | **Test Z** | **Significant** | **Test Z** | **Significant** | **Test Z** | **Significant** | **Test Z** | **Significant** |
| **January** | **-2.38** | ***** | **-5.25** | ***** | **6.26** | ***** | 1.48 |  |
| **February** | **-2.23** | ***** | **-5.22** | ***** | **6.91** | ***** | 1.95 |  |
| **March** | **-4.31** | ***** | **-4.92** | ***** | **6.55** | ***** | 1.09 |  |
| **April** | **-5.07** | ***** | **-3.75** | ***** | **5.90** | ***** | 0.16 |  |
| **May** | **-6.82** | ***** | **-5.05** | ***** | **4.23** | ***** | 1.48 |  |
| **June** | **-5.65** | ***** | **-4.12** | ***** | **3.53** | ***** | 0.62 |  |
| **July** | **-6.39** | ***** | **-4.42** | ***** | **2.64** | ***** | -0.93 |  |
| **August** | **-5.35** | ***** | **-5.13** | ***** | **4.26** | ***** | -0.93 |  |
| **September** | **-3.83** | ***** | **-3.88** | ***** | **5.05** | ***** | 1.56 |  |
| **October** | **-3.96** | ***** | **-6.05** | ***** | **6.11** | ***** | -0.86 |  |
| **November** | -1.43 |  | **-5.27** | ***** | **6.65** | ***** | -1.65 |  |
| **December** | **-2.53** | ***** | **-6.70** | ***** | **6.24** | ***** | 0.16 |  |

Table S5. Mann-Kendall trend analysis of monthly NO_2_, SO_2_, O_3_, and PM_2.5_ for Vancouver from 2000 to 2021. The test statistic Z represents the Mann-Kendall Z value, and the asterisk (*) indicates a statistically significant trend at the 95% confidence level.

| **Time series** | **NO_2_** | | **SO_2_** | | **O_3_** | | **PM_2.5_** | |
| --- | --- | --- | --- | --- | --- | --- | --- | --- |
|  | **Test Z** | **Significant** | **Test Z** | **Significant** | **Test Z** | **Significant** | **Test Z** | **Significant** |
| **January** | **-2.49** | ***** | -1.87 |  | -0.62 |  | -1.56 |  |
| **February** | **-2.96** | ***** | -1.87 |  | **2.65** | ***** | 1.65 |  |
| **March** | **-2.96** | ***** | **-3.27** | ***** | 0.62 |  | 1.71 |  |
| **April** | **-3.27** | ***** | **-2.18** | ***** | 0.31 |  | -0.62 |  |
| **May** | **-3.27** | ***** | **-2.48** | ***** | **2.34** | ***** | -0.47 |  |
| **June** | **-3.58** | ***** | **-1.56** |  | 0.62 |  | -1.34 |  |
| **July** | **-3.58** | ***** | **-2.18** | ***** | 0.62 |  | 1.64 |  |
| **August** | **-2.18** | ***** | **-1.71** |  | 0.31 |  | 0.16 |  |
| **September** | **-2.18** | ***** | **-1.87** |  | 1.25 |  | -1.09 |  |
| **October** | **-2.34** | ***** | **-2.34** | ***** | 1.09 |  | 1.49 |  |
| **November** | **-2.02** | ***** | **-2.18** | ***** | **2.49** | ***** | -1.87 |  |
| **December** | **-2.02** | ***** | **-2.49** | ***** | 1.87 |  | 0.62 |  |

Table S6. Mann-Kendall trend analysis of monthly NO_2_, SO_2_, O_3_, and PM_2.5_ for Calgary from 2000 to 2021. The test statistic Z represents the Mann-Kendall Z value, and the asterisk (*) indicates a statistically significant trend at the 95% confidence level.

| **Time series** | **NO_2_** | | **SO_2_** | | **O_3_** | | **PM_2.5_** | |
| --- | --- | --- | --- | --- | --- | --- | --- | --- |
|  | **Test Z** | **Significant** | **Test Z** | **Significant** | **Test Z** | **Significant** | **Test Z** | **Significant** |
| **January** | -0.47 |  | **-2.49** | ***** | 1.56 |  | 0.23 |  |
| **February** | **-2.71** | ***** | **-2.96** | ***** | 1.40 |  | 1.40 |  |
| **March** | **-2.56** | ***** | **-3.43** | ***** | **2.25** | ***** | 0.78 |  |
| **April** | -1.25 |  | **-3.11** | ***** | 0.78 |  | -1.71 |  |
| **May** | **-2.25** | ***** | **-3.11** | ***** | **2.56** | ***** | -0.39 |  |
| **June** | **-2.56** | ***** | **-3.27** | ***** | 0.93 |  | -1.40 |  |
| **July** | -1.09 |  | **-3.43** | ***** | 0.93 |  | 0.31 |  |
| **August** | -1.25 |  | **-3.27** | ***** | -0.16 |  | -0.47 |  |
| **September** | -1.09 |  | **-4.05** | ***** | 1.40 |  | -0.23 |  |
| **October** | -1.25 |  | **-3.43** | ***** | 0.16 |  | -1.96 |  |
| **November** | -0.78 |  | **-2.96** | ***** | 1.71 |  | 1.40 |  |
| **December** | -1.40 |  | **-3.11** | ***** | 1.71 |  | 0.47 |  |

Table S7. The Geographical characteristics of selected urban and rural stations.

| City | Station Name | Type | Latitude | Longitude | Elevation |
| --- | --- | --- | --- | --- | --- |
| Toronto | City Centre | Urban | 43.63 | -79.40 | 76.8 m |
|  | Georgetown | Rural | 43.64 | -79.88 | 221 m |
|  | Uxbridge West | Rural | 44.10 | -79.16 | 325 m |
|  | Mono Centre | Rural | 44.03 | -80.02 | 436 m |
| Montreal | MC Tavish | Urban | 45.5 | -73.58 | 72.80 m |
|  | Mirabel Intl | Rural | 45.67 | -74.03 | 82.30 m |
|  | ST-Hubert | Rural | 45.52 | -73.42 | 27.40 m |
| Vancouver | Harbour Centre | Urban | 49.30 | -123.12 | 2.5 m |
|  | Merry Island | Rural | 49.47 | -123.91 | 7.20 m |
|  | Saturna Island | Rural | 48.78 | -123.04 | 24.5 m |
| Calgary | Cop Upper | Urban | 51.08 | -114.22 | 1235 m |
|  | Black Diamond | Rural | 50.71 | -114.15 | 1156 m |

Table S8. Evaluation of the GEOS-Chem PM_2.5_ simulations (from 2010 to 2019) against observations from the NAPS stations.

| **Metrics** | **Toronto** | **Montreal** | **Vancouver** | **Calgary** |
| --- | --- | --- | --- | --- |
| **Correlation (R)** | 0.46 | 0.58 | 0.48 | 0.52 |
| **Mean Bias Error (MBE)** [μg m^−3^] | 5.39 | 0.57 | 4.85 | -1.5 |
| **Root Mean Square Error (RMSE)** [μg m^−3^] | 4.22 | 3.97 | 4.07 | 3.48 |

Table S9. Seasonal mean of UHI intensity 20-year-period (2000-2019) based on difference of mean temperature in urban and rural stations.

| City | Urban and rural stations | DJF | MAM | JJA | SON |
| --- | --- | --- | --- | --- | --- |
| Toronto | City Centre & Georgetown | **1.71** | 1.41 | 1.27 | 1.51 |
|  | City Centre & Uxbridge West | **3.63** | 1.98 | 1.78 | 2.32 |
|  | City Centre & Mono Centre | **3.44** | 2.09 | 2.2 | 2.66 |
| Montreal | MC Tavish & Mirabel Intl | **1.82** | 1.51 | 1.67 | 1.78 |
|  | MC Tavish & ST-Hubert | **1.06** | 0.98 | 1 | 0.92 |
| Vancouver | Harbour Centre & Merry Island | -0.22 | 0.01 | **0.24** | -0.16 |
|  | Harbour Centre & Saturna Island | -0.49 | 0.2 | **1.06** | -0.24 |
| Calgary | Cop Upper & Black Diamond | **1.6** | 0.5 | 0.68 | 0.87 |

Table S10. Change percent of number of rainy and windy days in 2020 relative to reference period of each city.

| Change in the number of rainy days (%) | | | | | | | | | | | | |
| --- | --- | --- | --- | --- | --- | --- | --- | --- | --- | --- | --- | --- |
|  | Jan | Feb | Mar | Apr | May | Jun | Jul | Aug | Sep | Oct | Nov | Dec |
| Toronto | 17 | -28 | 72 | -34 | -7 | -37 | 3 | 1 | -21 | 33 | -29 | 6 |
| Montreal | 20 | -21 | 51 | -33 | -15 | -33 | -5 | 19 | -23 | 13 | -4 | -15 |
| Vancouver | -40 | -16 | -5 | 13 | 9 | 35 | -6 | -13 | -20 | -30 | 13 | 20 |
| Calgary | -22 | 11 | 11 | 26 | 65 | -64 | -23 | -56 | -66 | 122 | -35 | 70 |
| Change in the number of windy days (%) | | | | | | | | | | | | |
| Toronto | -22 | -23 | 2 | 24 | 14 | 10 | 33 | 27 | 8 | -22 | 0 | 26 |
| Montreal | -33 | -19 | 12 | -58 | -2 | -53 | 13 | -23 | -5 | -23 | -32 | -36 |
| Vancouver | 87 | -13 | -35 | -12 | 0 | 8 | 18 | -100 | -100 | -12 | 8 | -76 |
| Calgary | -28 | -79 | -383 | -72 | -133 | -104 | 6 | -258 | -50 | 28 | -35 | 3 |

Table S11. Mean of the PM_2.5_ concentrations during pre lockdown and lockdown periods of 2020 and several references periods.

| City | Year/Period | Mean of pre lockdown | Mean of lockdown | SEM of pre lockdown | SEM of lockdown |
| --- | --- | --- | --- | --- | --- |
| Toronto | **2020** | **7.34** | **5.92** | 0.13 | 0.17 |
|  | 2019 | 8.03 | 6.16 | 0.31 | 0.22 |
|  | 2015-2019 | 8.84 | 6.82 | 0.07 | 0.04 |
|  | 2010-2019 | 8.10 | 7.92 | 0.05 | 0.04 |
|  | 2000-2019 | 7.27 | 6.85 | 0.02 | 0.03 |
| Montreal | **2020** | **8.08** | **4.80** | 0.26 | 0.12 |
|  | 2019 | 7.91 | 5.01 | 0.21 | 0.12 |
|  | 2015-2019 | 8.20 | 5.67 | 0.07 | 0.04 |
|  | 2010-2019 | 8.67 | 6.34 | 0.05 | 0.04 |
|  | 2000-2019 | 10.19 | 7.1 | 0.02 | 0.04 |
| Vancouver | **2020** | **3.13** | **3.9** | 0.09 | 0.14 |
|  | 2019 | 3.33 | 3.62 | 0.11 | 0.20 |
|  | 2015-2019 | 3.31 | 3.90 | 0.02 | 0.04 |
|  | 2010-2019 | 4.09 | 4.70 | 0.03 | 0.04 |
|  | 2000-2019 | 3.83 | 4.03 | 0.01 | 0.01 |
| Calgary | **2020** | **5.8** | **5.01** | 0.31 | 0.12 |
|  | 2019 | 8.79 | 6.81 | 0.27 | 0.27 |
|  | 2015-2019 | 8.53 | 6.25 | 0.08 | 0.07 |
|  | 2010-2019 | 8.98 | 8.15 | 0.07 | 0.06 |
|  | 2000-2019 | 7.2 | 6.37 | 0.02 | 0.02 |

- Pre lockdown period: Jan 2 to March 15
- Lockdown period: March 16 to Jun 3
- SEM: Standard Error of the Mean
